# Conserved storage-carbohydrate metabolic modules are rewired during germination of *Trichoderma asperelloides* and other Sordariomycetes

**DOI:** 10.3389/ffunb.2026.1930613

**Published:** 2026-08-26

**Authors:** Bar Tenennbaum, Elizabeta Yakubovich, Yen-Wen Wang, Guoliang Meng, Soumya Moonjely, Frances Trail, Julius Ben Ari, Zheng Wang, Caihong Dong, Jeffrey Peter Townsend, Oded Yarden

**Affiliations:** 1 Department of Plant Pathology and Microbiology, The Robert H. Smith Faculty of Agriculture, The Hebrew University of Jerusalem, Rehovot, Israel; 2 Department of Biostatistics, Yale School of Public Health, New Haven, CT, United States; 3 State Key Laboratory of Microbial Diversity and Innovative Utilization, Institute of Microbiology, Chinese Academy of Sciences, Beijing, China; 4 Department of Plant, Soil and Microbial Sciences, Michigan State University, East Lansing, MI, United States; 5 Laboratory for Mass Spectrometry and Chromatography, The Robert H. Smith Faculty of Agriculture, Food and Environment, The Hebrew University of Jerusalem, Rehovot, Israel; 6 Department of Ecology and Evolutionary Biology, Yale University, New Haven, CT, United States; 7 Program in Microbiology, Yale University, New Haven, CT, United States; 8 Program in Computational Biology and Biomedical Informatics, Yale University, New Haven, CT, United States

**Keywords:** Bayesian network inference, central-carbon metabolism, comparative transcriptomics, conidial germination, mannitol metabolism, Sordariomycetes, trehalose, *Trichoderma*

## Abstract

Conidial germination requires rapid mobilization and reorganization of storage carbohydrates, yet the network architecture underlying this process remains poorly defined in filamentous fungi. Using quantitative GC–MS/MS profiling, we provide the first quantitative identification of major soluble sugar species across four germination stages of *Trichoderma asperelloides* T203 and compared them with four representative models in the Sordariomycetes (*Metarhizium anisopliae*, *Cordyceps militaris*, *Fusarium graminearum*, and *Neurospora crassa*). In *T. asperelloides*, mannitol was the most prevalent measured sugar in dormant conidia, declined sharply at polarity establishment, and partially recovered at later stages, while trehalose displayed a reciprocal increase and other sugars remained comparatively stable. Comparative analyses revealed distinct species-specific carbon storage strategies: Dormant conidia of *T. asperelloides*, *M. anisoplia*e, and *C. militaris* were mannitol-enriched, whereas in *N. crassa* and *F. graminearum* glucose was the most abundant; after germination onset, most species shifted toward glucose accumulation, but *T. asperelloides* uniquely transitioned from mannitol to trehalose dominance before partial re-accumulation of mannitol. Integration of sugar profiles with time-resolved RNA-seq and Bayesian network inference revealed conserved core interactions but also lineage-specific divergences in mannitol/trehalose-associated central-carbon modules that correspond to distinct nutrient and lifestyle strategies during early colonization. A focused analysis in *T. asperelloides* uncovered extensive stage-dependent transcriptional remodeling of metabolic-process genes and a mannitol-centered module involving *mpd1* and *mtd1* (encoding mannitol‐1‐phosphate 5‐dehydrogenase and mannitol dehydrogenase, respectively). Antisense-based knockdown of *mpd1* strongly reduced its transcript levels and led to stage-dependent upregulation of *mtd1*. However, these changes left mannitol content, soluble-sugar profiles, germination dynamics, and growth on mannitol essentially unchanged. Together, our comparative metabolic-network analysis shows that conidial mannitol and trehalose metabolism in *T. asperelloides* is embedded in a flexible, partially redundant central-carbon framework, and establishes this species as a tractable model for systems-level dissection of sugar metabolic regulation during early fungal development and colonization.

## Introduction

Many filamentous fungi produce conidia as asexual propagules for reproduction and dispersal. Typically, conidia are dormant and more stress-resistant than vegetative hyphae, enabling them to survive environmental exposure until conditions permit germination. Favorable conditions lead to a tightly regulated developmental transition that requires prompt integration of multiple environmental and metabolic cues, ensuring that germination is initiated only under conditions conducive to successful growth (Osherov and May, 2001; Kasuga et al., 2005; Hagiwara et al., 2016; Novodvorska et al., 2016; Baltussen et al., 2020; Polozsányi et al., 2021; Ijadpanahsaravi and Wösten, 2024).

To support viability during dormancy and protect against environmental stressors such as drought, heat, oxidative stress, and nutrient limitation, fungal conidia accumulate a diverse pool of compatible solutes and metabolic reserves, including lipids, amino acids, glycogen, soluble sugars, and polyols. Among these, carbohydrates such as glucose and fructose, the disaccharide trehalose, and sugar alcohols, such as mannitol, arabitol, and sorbitol, are especially important during conidial survival and germination (Fillinger et al., 2001; Davis et al., 2000; Ruijter et al., 2003; Baltussen et al., 2020). Polyols are widely distributed in fungi and function as reserve carbohydrates as well as compatible solutes involved in osmoregulation and stress adaptation (Jennings, 1985; Hult and Gatenbeck, 1978). The relative abundance of polyols varies among a few intensively studied fungal models, mostly yeast-form fungi, under restrictively defined developmental stages. However, the available data indicate that they form a dynamic reserve pool that supports dormancy, resilience to environmental fluctuations, and the rapid metabolic transition required for germination (Davis et al., 2000; Khosravi et al., 2015; Baltussen et al., 2020).

*Trichoderma asperelloides* T203 (T203), a well-established biocontrol strain originally isolated in the 1980s (Sivan and Chet, 1989), is now recognized as a cryptic sister species of the metabolic model *T. asperellum* (Samuels et al., 2010; Steindorff et al., 2026). At the same time, based on the presence of an auxiliary gene that provides *T. asperelloides* with halotolerant attributes, Ding et al. (2026) demonstrated the ecological and physiological differentiation of this species within the *T. asperellum* complex. T203 has been extensively studied as a mycoparasitic fungus model, with documented fungal host range, host-recognition mechanisms, and hyphal-level interactions with phytopathogenic fungi (Elad et al., 1983; Sivan and Chet, 1989; Viterbo et al., 2002; Schuster and Schmoll, 2010). In addition, T203 also interacts beneficially with plants by promoting root colonization, transiently suppressing the plant immune responses, enhancing growth, and improving tolerance to salt stress (Shoresh et al., 2005; Viterbo and Horwitz, 2010). More recently, genome and transcriptome resources have expanded the use of T203 from a biocontrol model to a system for studying the developmental biology of conidial germination with comparative strategies among filamentous fungi with various lifestyles, suggesting a tight correlation between the rates of expression evolution and gene conservation (Wang et al., 2025). A comprehensive transcriptomic analysis of synchronous germination revealed extensive stage-dependent transcriptional reprogramming, including a broad decrease in transcript abundance during the transition from dormancy to establishment of polarity and substantial upregulation during later germination stages (Gortikov et al., 2022b; van Leeuwen et al., 2013). However, there is still a significant gap in understanding the primary metabolic processes during this critical morphological and ecological transition, which initiates growth and infection in filamentous fungi, including most medically and economically important species. Thus, T203 can serve as a useful model for examination of how dormant conidia transition to actively growing hyphae, supported not only by transcriptional remodeling, but also by the mobilization and reorganization of stored metabolic reserves.

**Figure 1:**
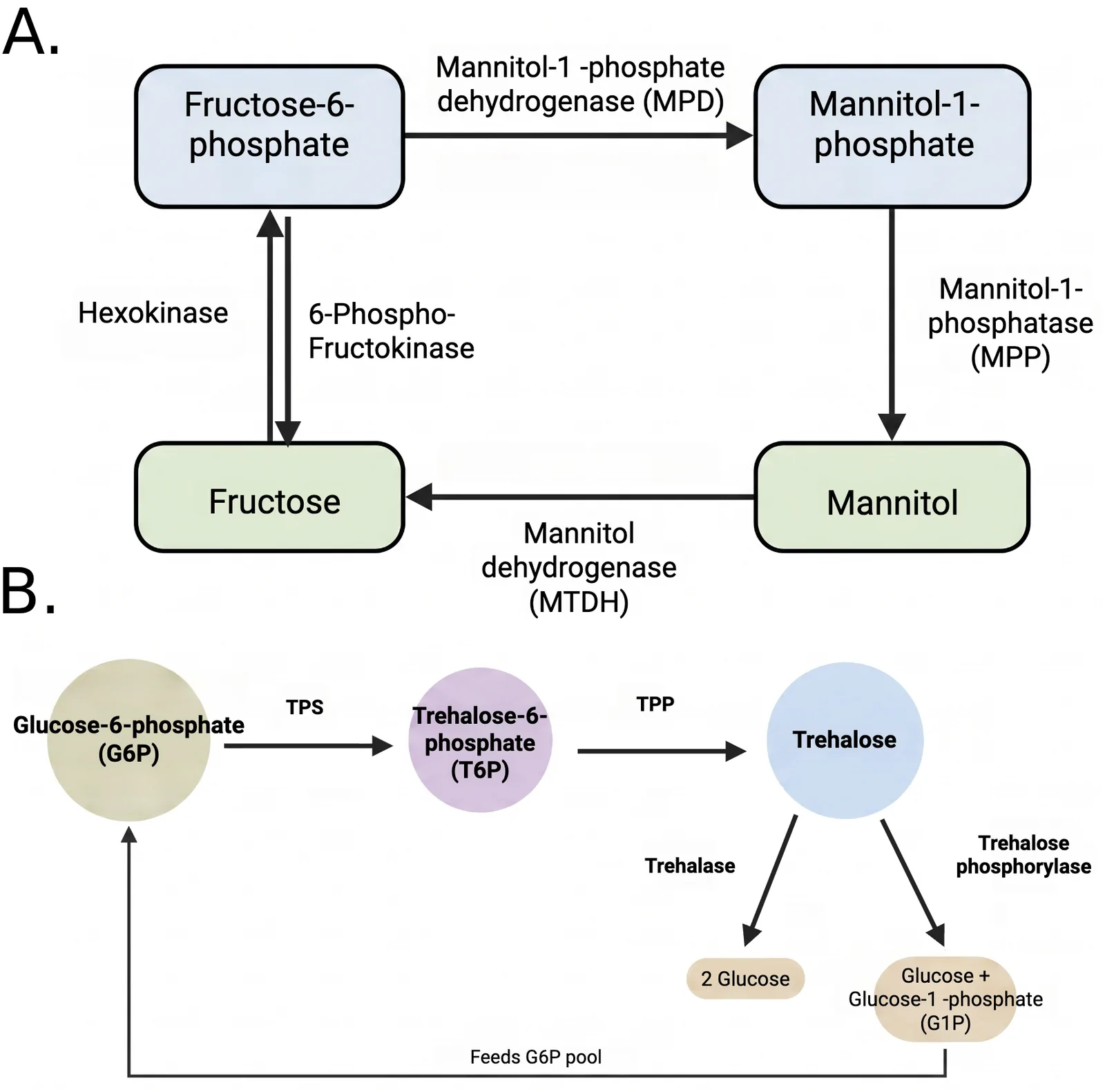
Putative trehalose and mannitol metabolism cycles in fungi. **(A)** Mannitol metabolism: MPD, mannitol phosphate dehydrogenase; MPP, mannitol phosphate phosphatase; FPP, fructose phosphate phosphatase; MTD, mannitol dehydrogenase; HX, hexokinase. **(B)** Trehalose metabolism: TPS, trehalose-6-phosphate synthase; TPP, trehalose-6-phosphate phosphatase.

Among stored metabolic reserves in fungi, mannitol is one of the most common polyols and is found in large quantities in spores, fruiting bodies, sclerotia, and mycelia. It plays several roles in fungal cells, such as maintaining osmoregulation, regulating coenzymes, quenching reactive oxygen species (ROS), providing reducing power, and a storing energy (Vélëz et al., 2007). In *Aspergillus niger*, for example, mannitol is rapidly metabolized during spore germination (Ruijter et al., 2003). In *T. atroviride*, mannitol has also been found in conidia, mycelia, and early germ tubes (Kaliňák et al., 2014). Studies have shown that dormant conidia of *Trichoderma* spp. accumulate high levels of mannitol, which sharply decline during the onset of polar growth before partially reaccumulating later (Kaliňák et al., 2014). The mannitol cycle (**Figure 1A**) was proposed more than 40 years ago based on studies in *Alternaria alternata* (Hult and Gatenbeck, 1978) that suggested the presence of two potential pathways. However, in most ascomycetes, only one primary cycle is recognized.

In this single cycle, fructose‐6‐phosphate (F6P) is converted to mannitol‐1‐phosphate (M1P) by mannitol‐1‐phosphate 5‐dehydrogenase (MPD; EC 1.1.1.17), and M1P is then dephosphorylated to mannitol, a function traditionally attributed to hexokinase activity (Voegele et al., 2005) (**Figure 1B**). In contrast, mannitol dehydrogenase, MTD, (EC 1.1.1.255) has been relatively underexplored and poorly characterized in fungi, with biochemical assays proving to be challenging, due to low enzyme activity. The role of MTDs as putative catabolic counterparts, oxidizing mannitol back to fructose to complete the redox-shuttling cycle, has emerged more clearly in later molecular and genetic studies (Stoop and Mooibroek, 1998; Voegele et al., 2005; Solomon et al., 2007).

Recent work in *Botrytis cinerea* has refined this picture and invokes questions as to whether mannitol metabolism in filamentous fungi operates as a single closed cycle. Targeted deletion of genes encoding the *B. cinerea* MPD1 and MTDH, combined with *in vivo* NMR flux analysis, revealed that glucose and fructose are preferentially routed through the MPD branch as well as the MTD branch. In turn, this dual routing implies that mannitol can be converted back to M1P via a previously unrecognized mannitol phosphorylation pathway. Strikingly, single and double Δ*mpd1*/Δ*mtd1* mutants still synthesized and degraded mannitol, and were able to grow on mannitol as the sole carbon source, indicating that additional mannitol-related enzymes and routes must contribute to carbon flow (Dulermo et al., 2010). Together, these data suggest that rather than a simple bidirectional redox cycle, fungal mannitol metabolism is organized as a more flexible, multi-branched network that integrates carbon storage, osmotic stress responses, and host-derived hexose sequestration.

In addition to the polyol mannitol, the disaccharide trehalose is commonly found in fungi, as well as being present in many other organisms, especially plants and bacteria. Trehalose serves as a survival and stress-tolerance metabolite in fungal spores and mycelium, and functions as a storage carbohydrate that is mobilized when cell growth resumes from a resting state (Fillinger et al., 2001; Treseder and Lennon, 2015). Trehalose not only stabilizes proteins and membranes under heat, osmotic, and oxidative stresses, but also has been shown to be essential for virulence in *Aspergillus nidulans* and *Candida albicans* among others (Thammahong et al., 2017; Lim et al., 2021). In ascomycetes, trehalose is predominantly synthesized via the canonical trehalose synthase complex (**Figure 1B**): a glucosyl moiety is transferred from UDP-glucose to glucose-6-phosphate (G6P) by trehalose-6-phosphate synthase (TPS; EC 2.4.1.15) to form trehalose-6-phosphate (T6P), which is then dephosphorylated to form trehalose by trehalose-6-phosphate phosphatase (TPP). A secondary pathway in which trehalose is synthesized from glucose-1-phosphate and glucose has been identified in basidiomycetes, and is thought to exist in species such as *Neurospora crassa* and *Aspergillus* spp (Wyatt et al., 2014; Galagan et al., 2003). Both the mannitol and trehalose cycles are linked to glycolysis, facilitating rapid metabolic response to environmental changes.

Despite advances in fungal genomics and metabolite profiling, the dynamic regulation and functional redundancy of storage carbohydrate metabolism during conidial germination is not completely understood. Recent studies highlight that transcript levels and metabolic flux can be decoupled due to post-transcriptional regulation, enzyme efficiency, or pathway compensation (Li et al., 2022). In this study, we use *T. asperelloides* T203 as a metabolic model to investigate how storage carbohydrate pools and associated transcriptional programs are reorganized during conidial germination and how these dynamics compare with those of four reference Sordariomycetes at distinct phylogenetic positions exhibiting distinct lifestyles: the entomopathogens *Metarhizium anisopliae* and *Cordyceps militaris*, the plant pathogen *Fusarium graminearum*, and the saprophyte *Neurospora crassa* (Gao et al., 2011; Zheng et al., 2011; Wang et al., 2019). We combined near-synchronous germination assays with GC-MS/MS sugar profiling, time-resolved RNA-seq, and Bayesian network analysis to: (i) characterize stage-specific remodeling of mannitol, trehalose, and other soluble carbohydrates in *T. asperelloides*; (ii) compare mannitol- and trehalose-associated central-carbon modules across Sordariomycetes with contrasting lifestyles; and (iii) test the functional contribution of the mannitol-1-phosphate dehydrogenase gene *mpd1* to conidial mannitol accumulation and germination. Our results reveal pronounced, stage-specific shifts in mannitol and trehalose pools, identify a conserved but partially rewired central-carbon module, and show that partial reduction of *mpd1* expression leaves conidial mannitol content and germination dynamics largely unchanged, suggesting substantial flexibility and redundancy in mannitol-associated carbon flux during germination.

## Materials and methods

### Strains, media, and culturing conditions

*Trichoderma asperelloides* strain T203 (Gortikov et al., 2022a) was obtained from the collection of the Department of Plant Pathology and Microbiology and was routinely cultured on potato dextrose agar or in potato dextrose broth (PDA or PDB, respectively; Difco Laboratories, Detroit, MI, USA) at 28 °C. *Cordyceps militaris* strain CGMCC 3.16323, from the China General Microbiological Culture Collection Center, Beijing, Chinese Academy of Sciences, was maintained on PDA at 20 °C. *Neurospora crassa* strain 74-OR23–1 from the Fungal Genetic Strain Center (FGSC#2489) was cultured and maintained as previously described (Wang et al., 2019). *Metarhizium anisopliae* (strain ARSEF549) and *Fusarium graminearum* (PH-1) were from the collection of the Department of Plant, Soil and Microbial Sciences (Michigan State University).

### Preparation of conidial suspensions

Conidia of the different fungi were harvested with 5 mL of cold double-distilled water (DDW), and gentle agitation with 4-mm glass beads. The resulting suspension was gauze filtered and centrifuged at 4,000 × *g* for 5 min at 4 °C. The pellet was washed twice with 30 mL of cold DDW. For preparation of near-synchronized germinating conidia, suspensions were stored overnight at 4 °C prior to inoculation. Approximately 1 × 10^5^ conidia were spread onto cellophane-overlaid PDA plates and incubated in constant darkness at 28 °C to monitor germination. Samples were collected at the relevant time points upon reaching the phase of (i) most (>90%) germinating conidia had reached or passed polarity establishment (roughly 7 hours), (ii) most (>90%) germlings had produced germtubes that were approximately twice the original conidial long-axis length (roughly 9 hours), and (iii) most (>90%) germlings exhibited first hyphal branching (roughly 12 hours). Samples collected at different stages were freeze-dried for sugar analysis.

### GC-MS/MS analysis of mono and disaccharides

Standards of arabinose, glucose, galactose, fructose, lactose, mannose, myo-inostilol, maltose, ribose, rhamnose, sucrose, trehalose, manitol, sorbitol, xylose, xylitol, and derivatization reagents, namely pyridine, trimethylchlorosilane (TMCS), and bis(trimethylsilyl)acetamide (BSA), were purchased from Sigma-Aldrich. Labeled 13C6 glucose, fructose, and sorbitol were purchased from Cambridge Isotopes. Calibration solutions of sugars were prepared in methanol/water (1:1) at the following concentrations: 0.5, 1, 2, 5, 10, 25, 50, 100, and 200 µg mL^-1^. The final concentration of internal standards (IS) used in experimental samples was 5 µg mL^-1^.

Fungal samples were lyophilized and ground in a homogenizer. 15-mg samples were extracted with 1.5 mL of water/methanol (1:1). Extraction was assisted by vortexing and sonication. After centrifugation at 11,200*g*for 10 min, 100 µL of the extract was transferred to a GC vial and spiked with a mixture of isotopically- labeled internal standards at a final concentration of 5 µg sample^-1^, evaporated to dryness, and chemically derivatized with hydroxylamine, followed by silylation using BSA/pyridine/TMCS reagent.

Analysis of mono- and disaccharides was carried out using a GC-MS/MS instrument that consisted of a gas chromatograph Trace 1310, mass selective detector TSQ 9000, and TriPlus RSH autosampler (all Thermo Scientific). The compounds were separated on a BPX-5 capillary column (30 m × 0.22 mm, 0.25 µm, SGE) using hydrogen as the carrier gas upon the following temperature gradient: initial temp 130 °C for 0.5 min, then to 195 °C at 8°/min, then to 204 °C at 3°/min, and finally to 290 °C at 12°/min (maintained for 6 min). The mass spectrometer was operated in EI MRM mode. The instrument was controlled using Xcalibur, and the data were analyzed by TraceFinder 5.1 software. A representative GC-MS/MS chromatogram of the analyzed sugars and MRM parameters is presented in **Supplementary Figure 1**.

### RNASeq analysis

RNA sequencing data for the different fungi were collected as part of previous analyses of genome-wide gene expression regulation during conidia germination in response to different environmental settings (Gortikov et al., 2022b; Wang et al., 2022, 2025; Moonjely and Trail, 2024). Differential transcript dynamics across germination stages were analyzed using the LOX framework (Wang et al., 2023; Zhang et al., 2010). LOX-derived expression profiles were then used to examine genes associated with mannitol, trehalose, glycolysis, and central carbon metabolism, and to guide the selection of candidate genes for RT-qPCR validation and Bayesian network analysis.

### Isolation of nucleic acids and RT-PCR

For RT-PCR, RNA was isolated from *T. asperelloides* cultures after freezing in liquid nitrogen and grinding to a powder using a mortar and pestle, using the TRIzol reagent and the 1- Bromo-3-chloropropane (BCP, Sigma Aldrich) method, as previously described (Herold et al., 2021). RNA samples were further purified with the RNA Clean and ConcentratorTM-5 kit (Zymo Research, Irvine, CA, USA) and treated with DNase (RNase-Free DNase Set, Qiagen, Hilden, Germany) according to the manufacturer’s instructions. RNA yield and quality were assessed using the Synergy™ HTX Multi-Mode Reader (Biotek, Winooski, VT, USA) and verified using gel electrophoresis. For RT-PCR, RNA was reverse transcribed using the High-Capacity cDNA Reverse Transcription kit (Applied Biosystems, Waltham, MA, USA). Transcript abundance was determined using a Step One Plus RealTime PCR (Applied Biosystems, Waltham, MA, USA) apparatus using gene-specific primers (**Table 1**) under standard reaction conditions as previously described (Gortikov et al., 2022b). To estimate the relative transcription in each sample, we calculated 2^-ΔΔCT^, where ΔΔCT is the ΔCT of the test sample and −ΔCT of the calibrator sample, and ΔCT is the difference between the cycle threshold (CT) of the gene of interest and the reference gene. Translation elongation factor 1α (*tef1α*) was used as a reference gene.

**Table 1:** Primers used for RT-PCR analyses.

Gene	Primer designation	Sequence (5’→3’)
Translation elongation factor 1α	Tef1α-F	TGACGGCAACCCCACTATCG
	Tef1α-R	CGAGTTCGGCGGCTTCCTAT
Mannitol-1-phosphate 5-dehydrogenase (KAH8125028)	MPD-F	AGTCCTCCACAACTCGGGCTAT
	MPD-R	TTGATGGCGCGGTAGTTT
Mannitol dehydrogenase (KAH8130487)	MTD_1-F	GTCGAGCGCTTTGTGAACCA
	MTD_1-R	CTTGAAGTGCGCACCAACG

### Plasmid construction

Plasmid pLY-4 was constructed on a pBSK backbone (Alting-Mees and Short, 1989) with an *amdS* gene cassette from the p3SR2 plasmid (Hynes et al., 1983; Pe'er et al., 1991). The cassette was flanked by 500 bp of upstream and downstream DNA of *mpd1* by restriction ligation. pLY-5 was cloned using the NEB HiFi Assembly kit. A synthetic insert containing the *A. nidulans trpC* promotor and an *amdS* gene cassette (TWIST Biosience, USA), was cloned into pBSK- for generation of *mpd1RNAas1 and mpd1RNAas2* mutants. Primers used for producing constructs are listed in **Table 2**. DH5α *E. coli* competent cells were prepared by the standard method of CaCl_2_ treatment and plasmid DNA was transformed following a Heat-Shock transformation procedure (Biolabs, Jerusalem, Israel). All DNA was extracted using a QIAprep Spin Miniprep Kit (QIAGEN, Hilden, Germany). PCR procedures were conducted using the BIO-RAD T100 thermal cycler (BIO-RAD, CA, USA). PCR amplifications were carried out using the Phusion^®^ High-Fidelity PCR Master Mix (New England BioLabs). pLY-4 plasmid construction was validated by DNA sequencing using native T3 and T7 primers (Hy Laboratories Ltd., Rehovot, Israel). pLY-5 construction was validated by primer-free whole-plasmid sequencing by Plasmidosaurus (Eugene, OR, USA). All PCR products during plasmid construction were purified using the Wizard SV gel and PCR Clean-Up System (Promega, Madison, WI, USA) using the manufacturers protocol.

**Table 2:** Primers used in this study for cloning.

Gene/DNA sequence	Primer designation	Sequence (5’→3’)
Acetamidase	amdS-F	CTCAATCCTGGGAAGAACTGG CAACGTCCTCTCTTCACTGAGTC
	amdS-R	
*mpd1* upstream	pLY-4_F1-F	TTCCCACACTCGCCGAGGCAGACCCTGTCCGGCCCTCTGA CCAAGCTCGAAATTAACCCTCACTAAAGGGATTGCGGTTTAT AAGGCAAAGA
	pLY-4_F1-R	
*mpd1* downstream	pLY-4_F2-F	GTGGATCCCCCGGGCTGCAGGACTCCCAAAGCAAGCTGCA
	pLY-4_F2-R	AGCCATGGGGATATACCCAAGTAGAAGTTAGGGATGGATGC AAAGGTT
Flanks+ *amdS* cassette	T3-F	GCGCAATTAACCCTCACTAAAG
	T7-R	GCTAGTTATTGCTCAGCGG
Flanks (500bp) + *amdS* cassette	pLY2_F_1 -F	CGCTGCCGGCACCTGTCCTACGCCAGCAGCACCTCTGCCA
	pLY2_F_2 -R	TTTAGAAAAATAAACAAATAGGGGTTCCGCGGAAGCACACG ATGTTTTGATTGC
ptrpC-*mpd1* antisense	pLY5 -F	CCAGAAGATGATATTGAAGGAGCA
	pLY5 -R	TGGATCCCGGTCGGCATCTACTGCCGAGGCTTCGTTGCTT

### Protoplast transformation of *T. asperelloides*

Protoplasts were obtained from *T. asperelloides* T203 germlings by modifications of the methods described by Pe'er et al. (1991), and Gortikov et al. (2022b). Briefly, conidia were inoculated into PDYC medium and incubated for 12 h at 28 °C with shaking at 150 rpm. The culture was centrifuged at 5,000 × g for 10 min, and the pellet was resuspended in a cell-wall-degrading enzyme solution containing 200 mg Vinotaste Pro (Novozymes, Denmark) and 400 mg Driselase (Sigma Aldrich) dissolved in 70 mL of 0.7 M NaCl. The suspension was incubated for 3 h at 28 °C with shaking at 70 rpm, until a protoplast concentration of approximately 1 × 10^9^ protoplasts mL^-^¹ was obtained. Protoplasts were filtered twice through sterile Miracloth and once through a 40-µm cell strainer, centrifuged at 4,500 × g for 5 min, washed once with cold 0.7 M NaCl and once with cold STC buffer [50 mM CaCl_2_, 10 mM Tris-HCl pH 7.5, and 1.2 M sorbitol], and finally resuspended gently in 200 µL cold STC buffer. Transformation was carried out using a PEG-mediated procedure. For each transformation, 10 µg of DNA, in a maximal volume of 25 µL, were mixed with 100 µL of protoplast suspension and incubated on ice for 12 min. Two sequential additions of 200 µL cold PEG 60% solution (0.5 g mL^-^¹ polyethylene glycol, 10 mM Tris-HCl pH 7.5, and 50 mM CaCl_2_) were performed, each followed by 5 min incubation on ice. Subsequently, 800 µL PEG 60% solution were added, and the mixture was incubated for an additional 6 min on ice. Finally, 1 mL cold STC buffer was added, and 1 mL of the transformation mixture was transferred to 40 mL regeneration buffer containing 1 g L^-^¹ yeast extract, 1 g L^-^¹ casein hydrolysate, and 1 M sucrose. Transformants were incubated overnight at 28 °C with shaking at 70 rpm. The following day, the transformation mixture was diluted 1:1 with cold 0.7 M NaCl, centrifuged at 4,500 × g for 10 min, washed sequentially with cold 0.7 M NaCl and cold STC buffer, and resuspended in 1 mL cold STC. Increasing volumes of the suspension, up to 500 µL, were mixed with warm acetamide selection medium and poured into Petri dishes. Plates were incubated at 28 °C until colonies appeared. Putative transformants were transferred to fresh acetamide selection medium and to PDA plates as a negative control. Molecular validation of the pLY5 transformant was performed by PCR using the primer pair pLY2_F_1/2-F/R (**Supplementary Figure 2**).

### Phylogenetic analysis

For phylogenetic analyses of the five sampled taxa, amino acid sequences of the RNA polymerase II subunits RPB1 and RPB2 [(Schoch et al., 2009; Wang et al., 2016) **Supplementary File 1**] were aligned using the MAFFT online service with default settings (Katoh and Standley, 2013; Katoh et al., 2019). The concatenated alignment of 3167 amino acids was used for Bayesian phylogenetic inference in MrBayes 3.2 (Ronquist et al., 2012) using the Metropolis-coupled Markov chain Monte Carlo (MCMCMC) algorithm with a mixed prior for the amino acid substitution model. Two independent analyses were run for 1,000,000 generations, with trees sampled every 1,000 generations. The first 25% of sampled trees were discarded as burn-in, and a majority-rule consensus tree was generated from the remaining trees. Clades with posterior probability (PP) > 0.98 were considered strongly supported. The tree was rooted with *Neurospora crassa* based on the current understanding of Sordariomycetes phylogeny (Maharachchikumbura et al., 2015; Hongsanan et al., 2017).

### Bayesian network prediction for functional genes groups during germination

Bayesian co-expression networks were modeled using the Bayesian Network Web Server (Ziebarth et al., 2013) to assess the regulatory associations among selected genes in the key metabolic pathways for stored carbohydrates during conidial germination among Sordariomycetes of various lifestyles. The predicted networks were based on expression data for each sampled species: *T. asperelloides, N. crassa, F. graminearum*, *M. anisopliae* and *C. militaris*, using orthologs involved in sugar metabolism, as inferred from our previous *T. asperelloides* dataset and comparative Sordariomycete germination transcriptomes (Gortikov et al., 2022b; Wang et al., 2025). Input files contained fold changes between adjacent sample points across the experiment (across four time points during conidial germination). Global structure learning settings were retained at default settings with four-tiered network structures. The models depicted are the 50% majority consensus of 100 models (selection threshold set to 0.5, the 100 highest-scoring networks were averaged), without imposing any structural constraints. Predicted network structure matrices were downloaded from the web server for further network divergence analysis. For JSD analysis, network matrices were generated from the averaged models. For the main comparative analysis, edges were retained using a posterior support threshold of 0.3. For comparative analysis across Sordariomycetes, homologous gene sets were identified in *N. crassa*, *F. graminearum*, *M. anisopliae*, and *C. militaris*. When several paralogs were present, the most likely orthologous representative was selected. Bayesian networks were inferred independently for each species using the corresponding transcript abundance data. The inferred networks were converted into structural matrices describing pairwise associations among genes. Prior to comparison, all matrices were reordered to the same gene order and symmetrized using an OR rule, in which an edge was retained if it appeared in either direction between a pair of genes. The symmetrized matrices were then flattened into matched vectors and compared pairwise using Jensen–Shannon divergence. The resulting JSD values were assembled into a distance matrix, which was used to construct a neighbor-joining tree representing similarity among species based on inferred regulatory-network structure.

### Statistical analysis

Statistical analyses were performed in Python version 3.12.2 using the SciPy/statsmodels package, version 1.15.3, 0.14.4. Technical RT-qPCR replicates were averaged prior to statistical analysis, and independent biological replicates were treated as the experimental units. Relative transcript abundance was calculated using the 2−ΔΔCt method and is presented as the mean ± standard deviation (SD). For the developmental expression analysis the effects of genotype, developmental stage, and their interaction were evaluated using two-way analysis of variance (ANOVA), followed by Dunnett multiple-comparisons test.

## Results

### Mannitol and trehalose dynamics accompany conidial germination of *T. asperelloides*

We first determined the abundance of major sugar species present in *T. asperelloides* conidia and monitored the changes occurring in sugar composition during the different stages of germination.

**Figure 2:**
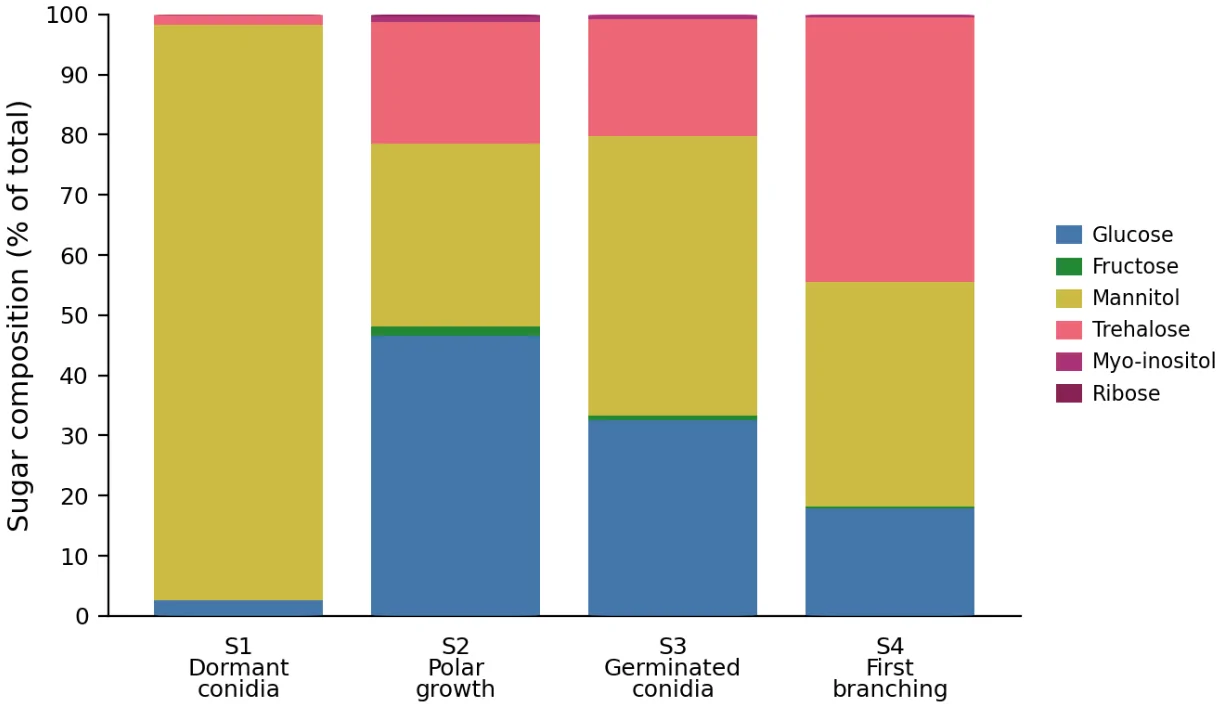
Relative abundance of key sugars found in *Trichoderma asperelloides* during germination. Sugars were extracted from cultures, grown in PDB in a near-synchronous manner, at 4 developmental stages: (S1) rehydrated conidia (S2) establishment of polarity, (S3) formation of germ tubes and (S4) first branching. Total values represent the sum weight of analyzed sugars and polyols at each germination stage from two biological replicates.

Among the sugars analyzed, mannitol was found to constitute about 75 percent of the total tested sugars and polyols in dormant conidia (**Figure 2**), making it the major component in this preliminary stage of germination. The relative amount of mannitol dropped to 13 percent at the polar growth stage - a 6-fold decrease compared to the initial amount. As conidia proceeded to the last two germination stages (germinated conidia and first branching), an increase in mannitol levels was measured and reached about 22 percent and 26 percent, respectively.

In contrast to mannitol, trehalose exhibited a different pattern of sugar utilization. The initial amount of trehalose in dormant conidia constituted only about 6 percent of the total analyzed components, which is about 13-fold less than that of the initial amount of mannitol in dormant conidia (**Figure 2**). As germination progressed, trehalose levels were found to increase to a relative amount of about 65 percent and 50 percent in germinated conidia and first branching stages, respectively. Levels of the tested soluble sugars including fructose, sorbitol, glucose, and myo-inositol did not show substantial changes along the four germination stages.

### Mannitol is the predominant conidial polyol in several Sordariomycete lineages

**Figure 3:**
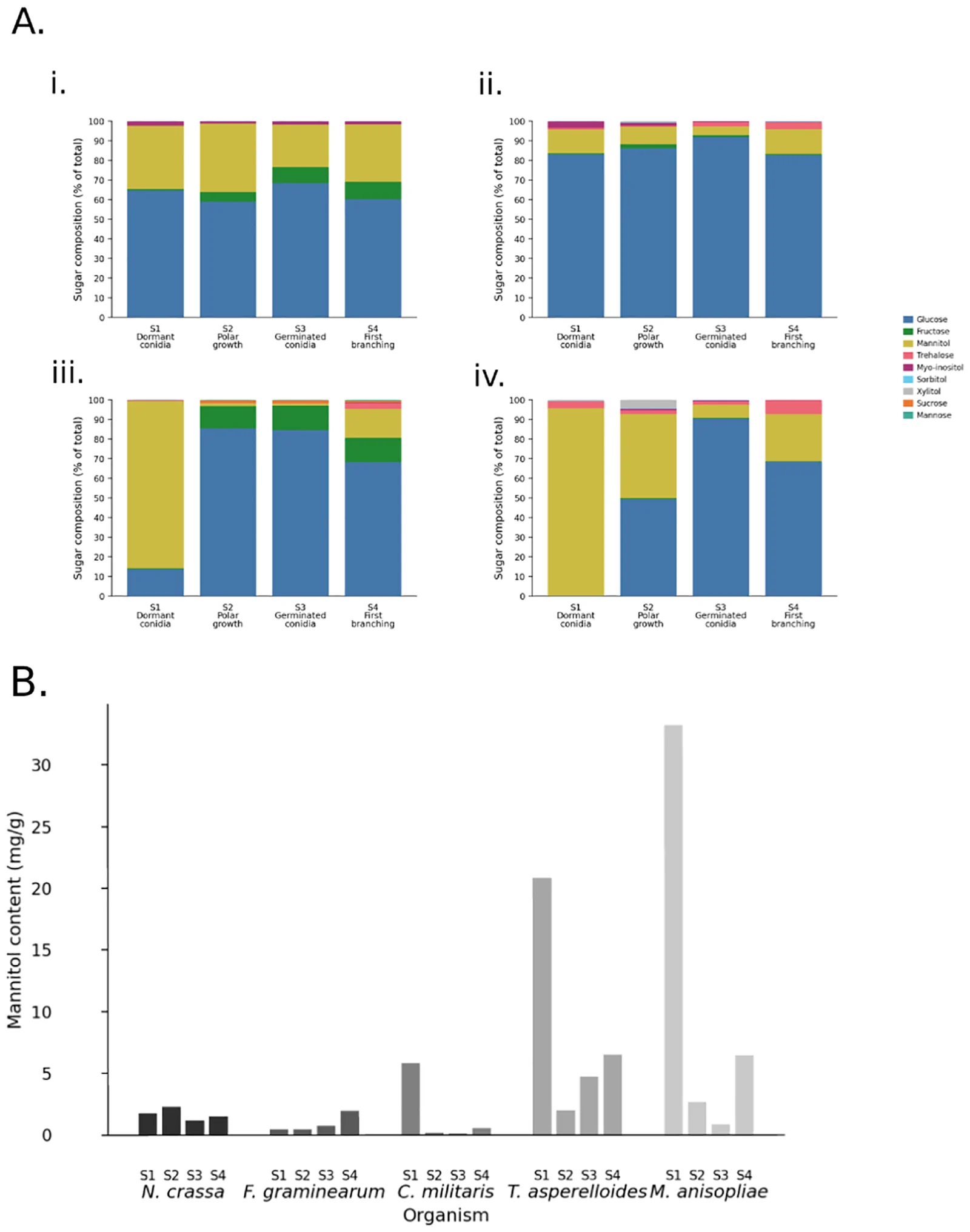
**(A)** Main sugar composition found in 4 Sordariomycetes reference species during gemination- (i)-*N. crassa*., (ii)-*F. graminearum., (*iii)- *C. militaris.*, (iv)- *M. anisopliae.* Sugars were extracted from cultures, grown on PDB in a near-synchronous manner, at 4 developmental stages: (S1) rehydrated conidia (S2) establishment of polarity, (S3) formation of germ tubes and (S4) first branching. **(B)** Mannitol (mg g^-1^ of sample dry weight) content of 5 sordariomycetes during different stages of germination.

Given the accumulated genomic and transcriptomic information available for members of the Sordariomycetes (Wang et al., 2023), we expanded our conidial sugar-composition analysis to four additional reference species representing distinct phylogenetic positions and lifestyles: the saprophyte *N. crassa*, the plant pathogen *F. graminearum*, and the insect-associated fungi *M. anisopliae* and *C. militaris*. Soluble carbohydrate and polyol dynamics were then followed across the corresponding stages of conidial germination (**Figure 3**).

In the more distantly related lineages, *N. crassa* and *F. graminearum*, glucose was the predominant measured carbohydrate in dormant conidia. In contrast, dormant conidia of *M. anisopliae*, *C. militaris*, and *T. asperelloides* were dominated by mannitol (**Figure 3A**). Following germination initiation, most of the reference fungi showed a shift toward glucose accumulation. In *T. asperelloides*, however, the mannitol-dominated dormant-conidial profile was followed by the distinct mannitol/trehalose redistribution described above, including partial mannitol recovery at first branching (**Figure 3B**). Thus, although high dormant-conidial mannitol was shared by several taxa, the subsequent carbohydrate/polyol remodeling was especially pronounced in *T. asperelloides* and *M. anisopliae*. Given this distinctive pattern, we next compared mannitol/trehalose-associated transcriptional networks across the five species to assess the extent to which the underlying metabolic-network structure was conserved despite divergent storage-carbohydrate dynamics.

### Multiple “metabolic process” GO-term classified genes undergo changes in expression during *T. asperelloides* conidial germination

Based on the observed dynamics in sugar composition during *T. asperelloides* conidial germination, we further examined transcriptional dynamics in genes associated with metabolic processes along with the morphological changes, with particular interest in pathways related to mannitol and trehalose metabolism. The global transcriptional dynamics of *T. asperelloides* during conidial germination, with emphasis on cell-wall remodeling, were previously described by Gortikov et al. (2022b). Using this dataset, we analyzed genes classified under the “metabolic process” GO term and compared their LOX-derived expression dynamics across three consecutive developmental transitions: dormant conidia to polar growth, polar growth to germinated conidia, and germinated conidia to first branching.

**Figure 4:**
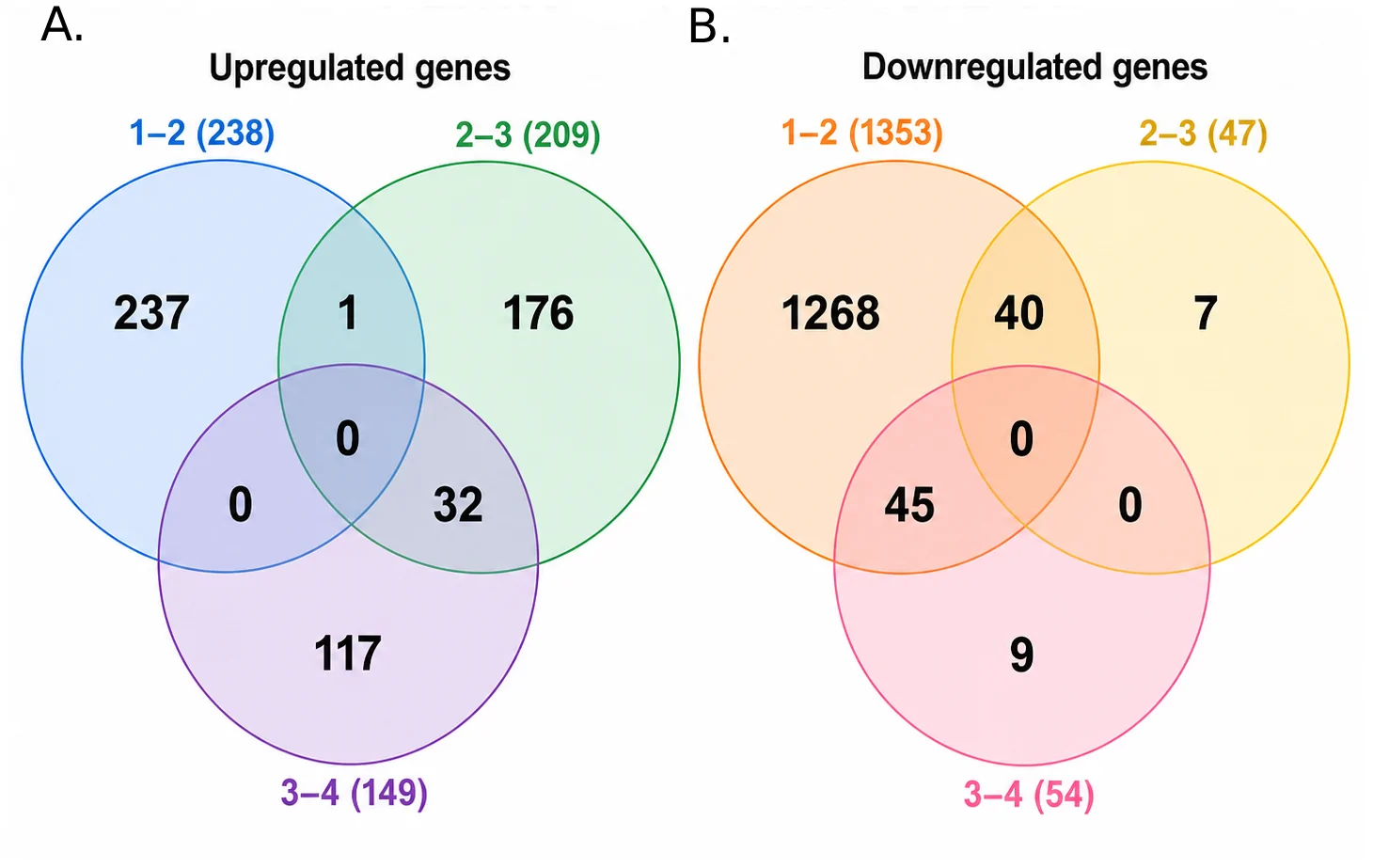
Previously published RNA-seq data from near-synchronous *T. asperelloides* conidial germination (Gortikov et al., 2022b) were reanalyzed using LOX differential-expression analysis, after which genes classified under the “metabolic process” GO term were selected. Venn diagrams summarize the numbers and overlap of significantly **(A)** upregulated and **(B)** downregulated genes across three developmental transitions: Stage 1→Stage 2, dormant conidia to polarity establishment; Stage 2→Stage 3, polarity establishment to germ-tube formation; and Stage 3→Stage 4, germ-tube formation to first branching. Differentially expressed genes were defined as having a fold change >2 for upregulated genes or <−2 for downregulated genes.

Marked transcriptional changes were observed during conidial germination in all examined species. In *T. asperelloides*, the most pronounced shift occurred during the transition from dormant conidia to polar growth, in which 238 genes were upregulated (**Figure 4A**) and 1,353 genes were downregulated (**Figure 4B**). During the transition from polar growth to germinated conidia, 47 genes were downregulated and 209 genes were upregulated. In the final transition, from germinated conidia to first branching, 54 genes were downregulated and 149 genes were upregulated. Most downregulated genes were specific to the first developmental transition. Of the genes downregulated between dormant conidia and polar growth, 1,268 genes were unique to this transition, while 40 genes were also downregulated in the following transition and 45 genes were also downregulated during the transition from germinated conidia to first branching. No downregulated genes were shared across all three developmental transitions. In contrast, the upregulated response was distributed more broadly across later stages: 237 genes were uniquely upregulated during the transition from dormant conidia to polar growth, 176 genes were uniquely upregulated from polar growth to germinated conidia, and 117 genes were uniquely upregulated from germinated conidia to first branching. Only one gene was shared between the first and second upregulated transitions, whereas 32 genes were shared between the second and third transitions. No upregulated genes were shared across all three comparisons.

### Gene expression and regulatory interaction between genes involved in mannitol/trehalose metabolism during germination

**Figure 5:**
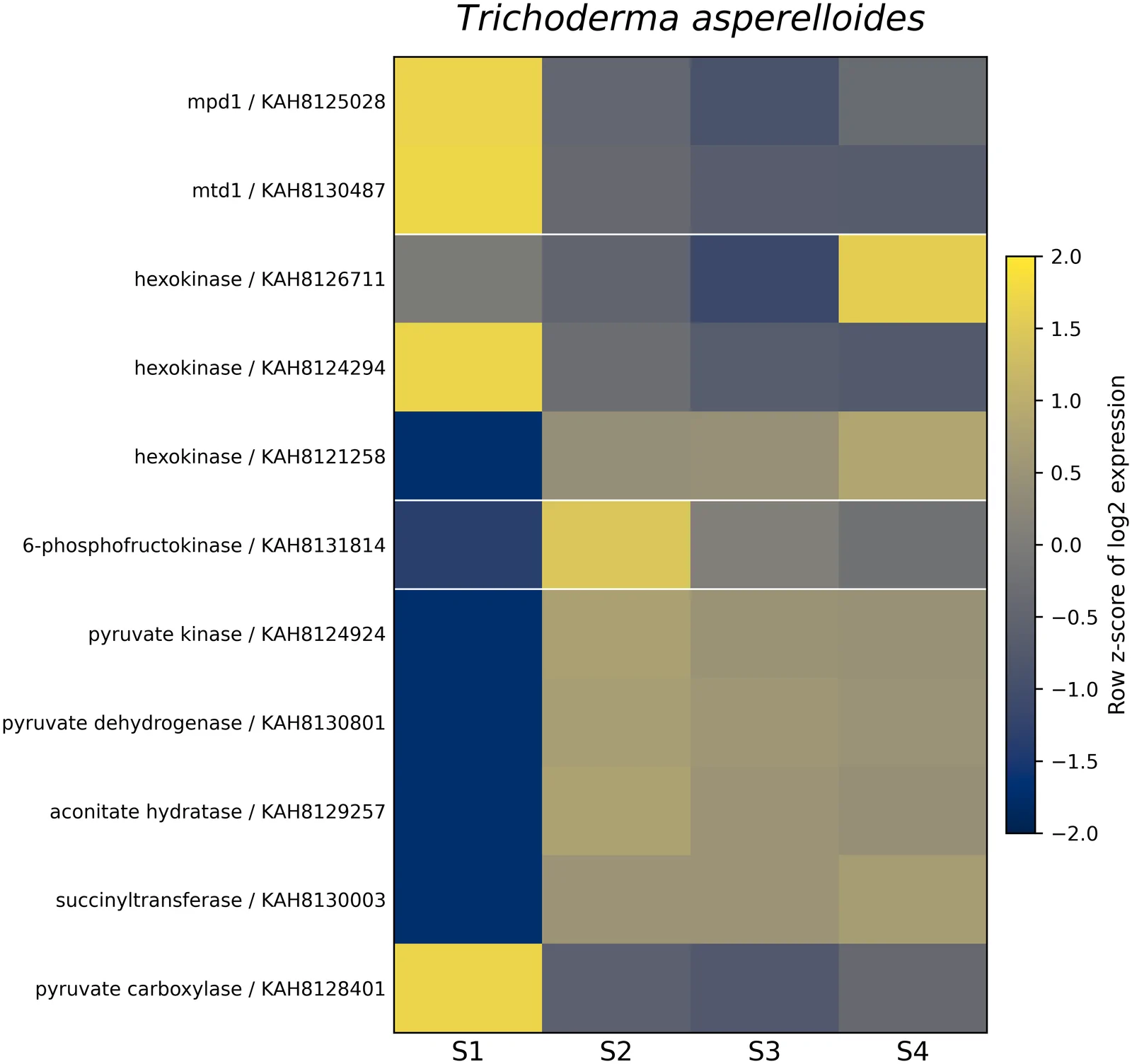
Expression dynamics of selected mannitol/trehalose and central-carbon metabolism genes during *T. asperelloides* conidial germination based on transcriptomic data previously reported by Gortikov et al. (2022b). Heatmap showing normalized transcript abundance of selected genes associated with mannitol metabolism, trehalose metabolism, glycolysis, and the TCA cycle across the four developmental stages analyzed in this study: dormant conidia, polar growth, germinated conidia, and first branching. Expression values were scaled per gene to emphasize developmental expression dynamics. The selected gene set includes *mpd1/man1*, *mtd1*, candidate hexokinases, *pfk1*, *pki1*, *pda1*, *ach1*, *sct1*, *pyc1*, and trehalose-associated genes. This focused expression analysis provides the basis for the subsequent Bayesian-network reconstruction of a mannitol-associated central-carbon regulatory module.

Among the selected genes, *mpd1/man1*, encoding a predicted mannitol-1-phosphate 5-dehydrogenase, showed a prominent developmental expression pattern, with highest transcript abundance in dormant conidia followed by a marked decrease during *T. asperelloides* germination. This pattern was consistent with the sharp decline in mannitol content observed during the transition from dormancy to polar growth and suggested that mannitol-associated genes may be embedded within a broader central-carbon transcriptional program. A focused heatmap of selected carbohydrate- and central-carbon-metabolism genes further illustrates the stage-dependent expression patterns of candidate genes used for subsequent RT-qPCR validation and network analysis (**Figure 5**). The heatmap revealed distinct clusters of genes with elevated transcript abundance at different phases of germination. Several genes showed higher expression in dormant or early polar-growth stages, consistent with the rapid mobilization of pre-existing storage reserves during germination initiation. In contrast, other genes were preferentially induced during later germination stages, particularly during germ tube formation and first branching, suggesting a transition from reserve mobilization toward renewed carbohydrate biosynthesis, glycolytic activity, and central-carbon-metabolism remodeling. Expression patterns of the same genes were also analyzed in the other Sordarimycetes. However, the single-gene heatmaps did not provide a consistent pattern (**Supplementary Figure 3**). Together, these results indicate stage-dependent transcriptional reprogramming of metabolic-process genes during *T. asperelloides* conidial germination. We therefore used a focused bioinformatic approach to examine potential regulatory associations among genes assigned to mannitol metabolism, glycolysis, and the TCA cycle. Bayesian network (BN) was inferred from LOX measurements of chosen genes from these groups and highlighted a regulatory hub composed of eight genes (**Table 3**). Albeit at a lower level than some of the other network components, the resulting network supports the interaction/dependency of *mdp* and *mtd1*.

**Table 3:** Selected KEGG pathways gene identification and annotation.

E.C number	Gene_ID	Annotation (By E.C number)	Designation	References
Fructose and mannose metabolism				
1.1.1.17	gene_6264	Mannitol-1-phosphate, 5-dehydrogenase	*mpd1*	This study
1.1.1.255	gene_2761	Mannitol dehydrogenase	*mtd1*	(Dulermo et al., 2010)
2.7.1.1	gene_4773	Hexokinase	*hxk1*	(Hartl & Seiboth, 2005)
	gene_6972	Hexokinase	*hxk1*	
	gene_8571	Hexokinase	*hxk1*	
2.7.1.11	gene_9544	6-phosphofructokinase	*pfk1*	This study
Glycolysis				
2.7.1.40	gene_5626	Pyruvate kinase	*pki1*	(Schindler et al., 1993)
TCA cycle				
1.2.4.1	gene_10448	Pyruvate dehydrogenase (acetyl-transferring)	*pda1*	This study
4.2.1.3	gene_11114	Aconitate hydratase	*ach1*	This study
2.3.1.61	gene_10662	Succinyltransferase (dihydrolipoyllysine-residue)	*sct1*	This study
6.4.1.1	gene_152	Pyruvate carboxylase	*pyc1*	This study

**Figure 6:**
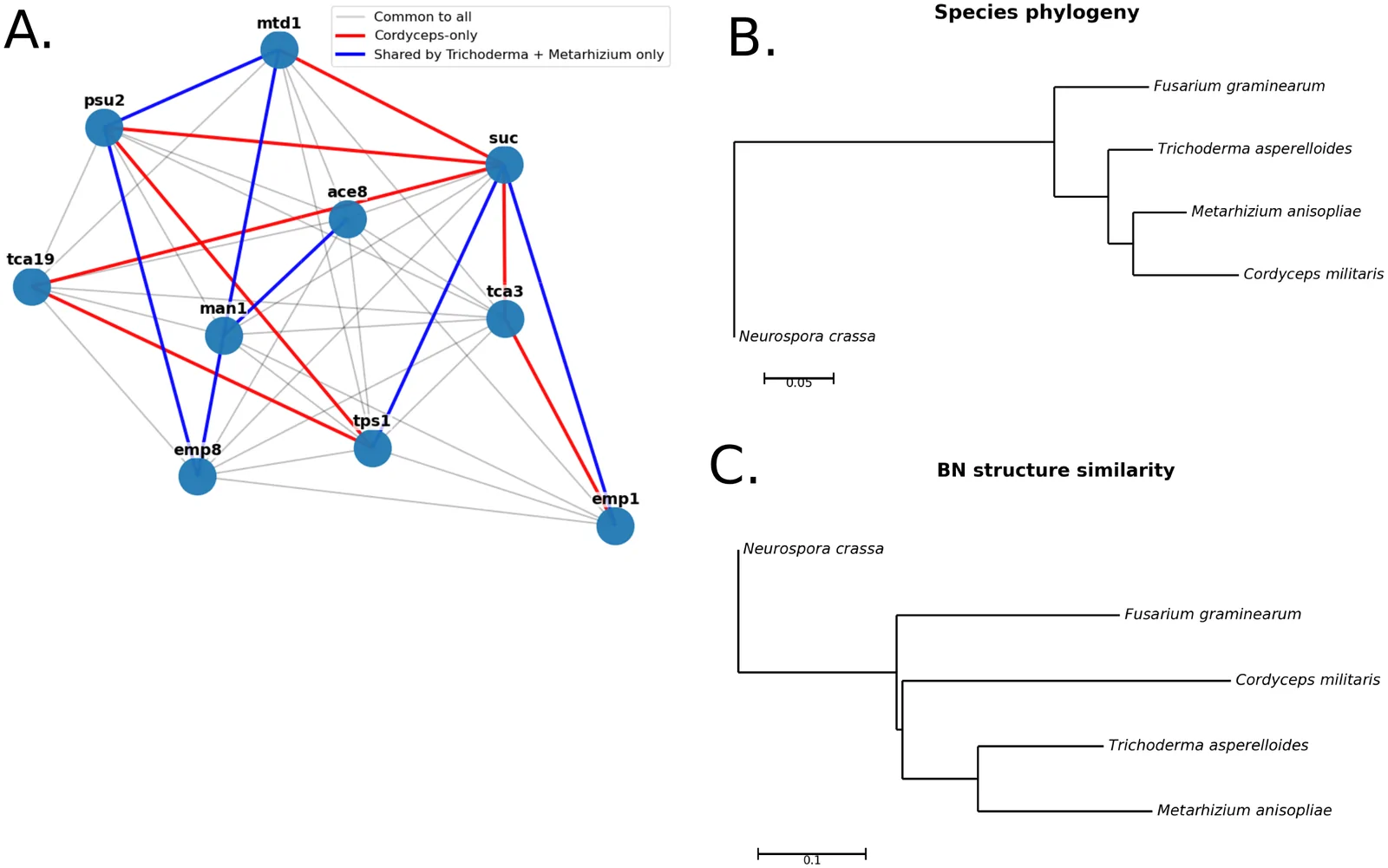
Comparative analysis of mannitol associated regulatory network structure across Sordariomycetes. **(A)** Comparative representation of the Bayesian-network-derived regulatory module comprising genes associated with mannitol metabolism, glycolysis, and the TCA cycle. Gray edges represent associations retained in all analyzed species, red edges represent *C. militaris*-specific associations, and blue edges represent associations shared specifically by *T. asperelloides* and *M. anisopliae*. **(B)** Bayesian species tree inferred from concatenated RPB1 and RPB2 protein sequences. Branch lengths and the scale bar represent the expected number of amino-acid substitutions per site. Values at internal nodes represent Bayesian posterior probabilities, where shown. **(C)** Neighbor-joining tree constructed from pairwise Jensen–Shannon distances calculated between symmetrized Bayesian-network structural matrices. Branch lengths and the scale bar represent Jensen–Shannon distance units. Shorter distances indicate greater similarity between network structures.

To examine whether the regulatory module identified in *T. asperelloides* reflects a broader evolutionary pattern, we compared the mannitol-associated network structure across representative Sordariomycetes (**Figure 6**). The analyzed gene set was derived from the preceding *T. asperelloides* RNA-seq/LOX analysis, in which genes associated with mannitol metabolism, glycolysis, and the TCA cycle formed a focused regulatory module. In the comparative network representation, several interactions were shared among all analyzed species, indicating conservation of core associations within this metabolic module (**Figure 6A**). However, additional interactions were species- or lineage-associated. Notably, *Cordyceps*-specific edges and edges shared specifically by *Trichoderma* and *Metarhizium* were detected, suggesting that the conserved metabolic framework has undergone partial rewiring in different Sordariomycete lineages.

To determine whether these network differences reflect phylogenetic relatedness or metabolic similarity, the inferred network structures were compared with the species phylogeny using Jensen–Shannon divergence (**Figures 6B, C**). A neighbor-joining tree constructed from the resulting JSD distance matrix revealed partial correspondence with the organismal phylogeny, but also showed clustering that may reflect similarities in storage-carbohydrate dynamics rather than phylogenetic distance alone (**Figure 6C**). In particular, the relatively close placement of *T. asperelloides* and *M. anisopliae* in the network-based tree is consistent with the presence of shared network edges (*mdp1*-*mtd1* among others) between these species.

### Reduced expression of Mannitol-1-Phosphate Dehydrogenase does not alter mannitol metabolism during germination of *T. asperelloides*

The high abundance of mannitol in dormant *T. asperelloides* conidia, together with its rapid depletion during polarity establishment, suggested that mannitol metabolism may contribute to the early physiological transition from dormancy to active growth. To determine to the extent to which the predicted mannitol-biosynthetic branch is required for normal conidial germination and associated carbohydrate remodeling, we targeted *mpd1*, which encodes a putative mannitol-1-phosphate dehydrogenase.

**Figure 7:**
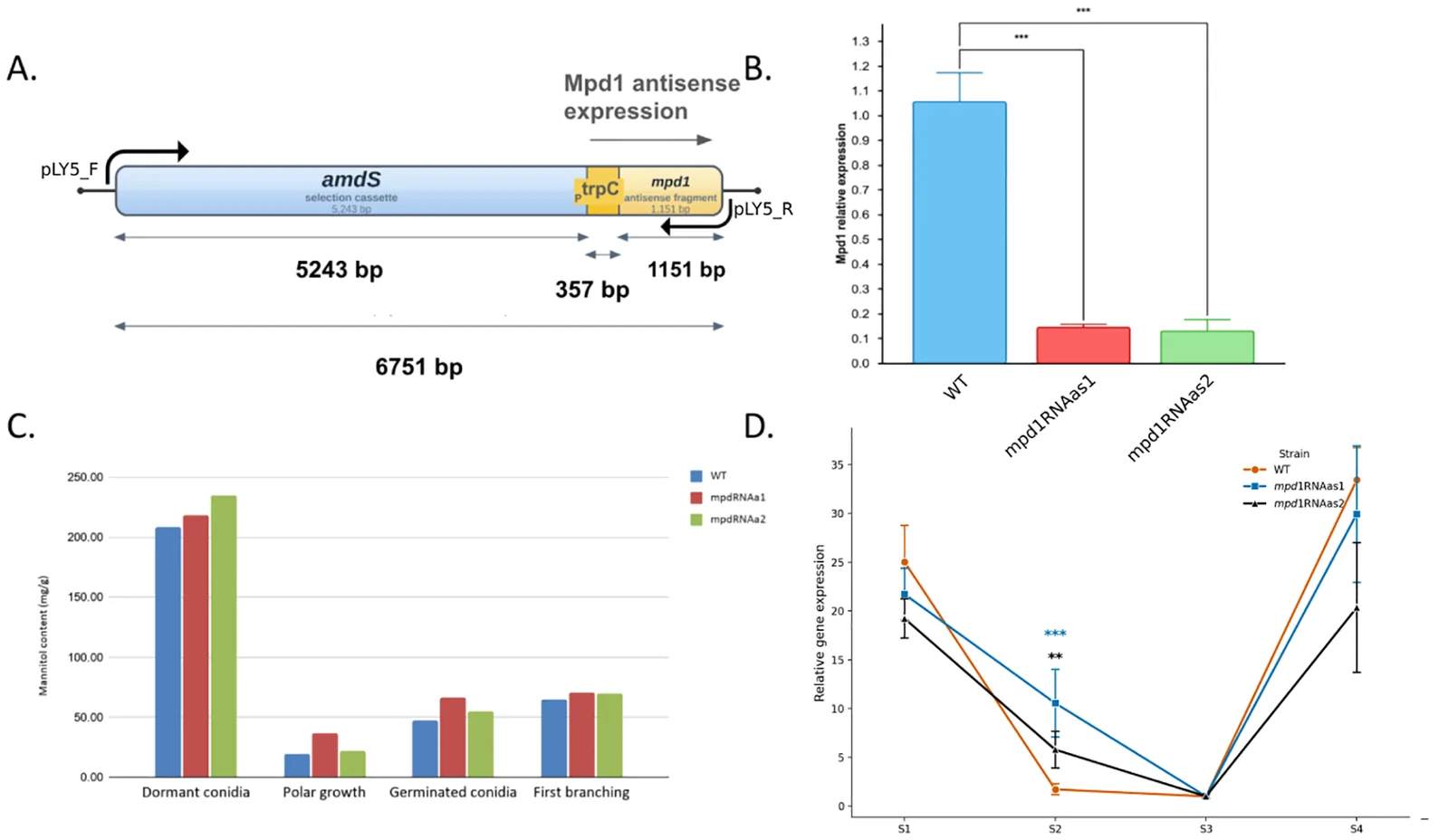
**(A)** Schematic representation of the *mpd1* antisense strategy used to generate *mpd1*-knockdown transformants. Arrows show the positions and orientations of the primers used for genomic-PCR screening of the transformants. **(B)** Quantitative RT-qPCR analysis of *mpd1* transcript abundance in the wild-type strain (WT) and two independent antisense transformants (*mpd1RNAas1* and *mpd1RNAas2*) at the rehydration stage. RNA was extracted from cultures grown in PDB under near-synchronous germination conditions. Relative transcript abundance was normalized to *tef1α*. Values represent means of three biological replicates; error bars indicate SD. Statistical analysis was performed in Python using two way ANOVA, followed by Dunnett multiple-comparisons test. Asterisks indicate statistically significant differences: ***P<0.01P. **(C)** Mannitol content measured by GC-MS in WT, *mpd1RNAas1*, and *mpd1RNAas2* across four germination stages: rehydration, polarity establishment, germ tube formation, and first branching. Mannitol content was also measured in rehydrated conidia of *mpd1RNAas1* grown on mannitol as the sole carbon source. Values are expressed as mg g^-1^ of sample dry weight. **(D)** Developmental expression profile of *mtd1* in the wild-type strain and two independent *mpd1* antisense lines, *mpd1RNAa1* and *mpd1RNAa2*, during rehydration, polarity establishment, germ-tube formation, and first branching. Values represent the mean ± SD of three independent biological replicates. Statistical analysis was performed in Python using two way ANOVA followed by Dunnett multiple-comparisons test. Asterisks indicate statistically significant differences: **P<0.05, ***P<0.01P.

Following transformation of T203 conidia with pLY-4, 37 transformants were screened by PCR. Only one candidate displayed evidence of homologous gene replacement. However, further analysis revealed this strain was a heterokaryon containing both the native *mpd1* allele along with the integrated cassette. Attempts to isolate homokaryotic transformants through conidial selection on acetamide medium and hyphal tip isolation were unsuccessful, as all colonies retained heterokaryotic or wild-type genotypes. Given these challenges, we employed an RNA antisense (RNAas) strategy to reduce *mpd1* expression. Two independent *mpd1* antisense RNA expressing mutants (**Figure 7A**), designated *mpd1RNAas1* and *mpd1RNAas2*, were selected for characterization. Quantitative RT-PCR analysis confirmed that *mpd1* transcript abundance was significantly reduced in these transformants relative to wild type (**Figure 7B**), across three biological replicates, validating the effectiveness and reproducibility of the RNA-antisense-based approach.

To assess whether decreased *mpd1* expression altered carbohydrate metabolism, we analyzed soluble sugar profiles at the four defined stages of germination (**Figure 7C**). Across all stages, mannitol remained the predominant soluble carbohydrate in both WT and RNAas strains during dormancy, decreasing sharply at polarity establishment and partially recovering at subsequent stages. Furthermore, no differences in concentration of other polyols and the additional sugars analyzed (trehalose, glucose, fructose, sorbitol, myo-inositol) were detected between RNAas transformants and WT at any stage (**Supplementary Figure 4**). Thus, partial reduction of *mpd1* expression did not substantially alter the overall carbohydrate composition during germination.

### Reduced expression of *mpd1* does not impair growth of *T. asperelloides* cultures grown on mannitol as a sole carbon source

Given the fact that mannitol-1-phosphate dehydrogenase (encoded by *mpd1*) catalyzes the reversible conversion between fructose-6-phosphate and mannitol-1-phosphate, and thus can play a central role in both the biosynthesis and catabolism of mannitol in filamentous fungi, we examined its requirement for *T. asperelloides* growth on mannitol as sole carbon source. Specifically, we cultured both wild-type and *mpd1* RNAas knockdown strains in minimal medium with mannitol provided as the sole carbon source—a condition that necessitates both the import and metabolic utilization of exogenous mannitol. GC-MS analysis of dormant conidia harvested from these cultures (rehydration stage) revealed that mannitol levels in the RNAas knockdown strain were indistinguishable from those in wild-type, even when *mpd1* transcript levels were severely reduced.

The complete soluble sugar and polyol profiles of WT and independent *mpd1RNAas1* isolates across all germination stages are provided in **Supplementary Figure 2**, indicating that the overall carbohydrate composition and developmental dynamics were broadly similar among isolates.

**Figure 8:**
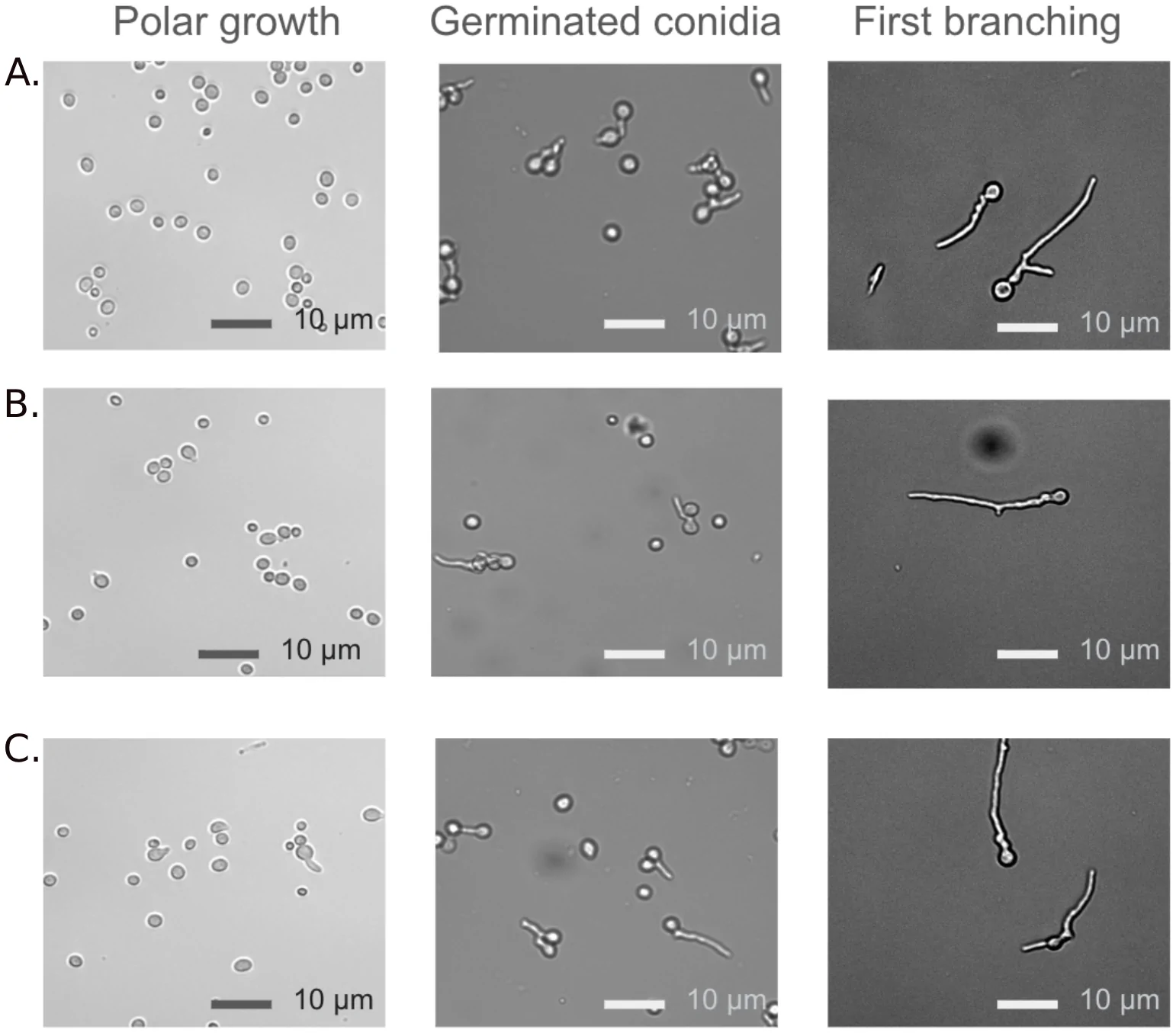
Germination morphology of WT and *mpd1RNAas* mutant conidia grown in PDB at 28 °C, 150 rpm. Representative images showing the three post-dormancy germination stages analyzed in this study: polar growth, germinated conidia, and first branching. WT **(A)** and independent *mpd1RNAas* **(B, C)** mutant isolates mutant isolates, which exhibited broadly similar morphological and developmental progression across all examined stages.

To determine whether the apparent robustness of mannitol accumulation in the *mpd1*-knockdown strains was accompanied by changes in the expression of an alternative mannitol-metabolic gene, we examined the developmental expression profile of *mtd1* by RT-qPCR (**Figure 7D**). In the WT, *mtd1* expression displayed a strongly stage-dependent profile, with high relative expression in dormant conidia and again at the first-branching stage, while expression was lowest at the germinated-conidia stage. In contrast, both *mpd1*-knockdown lines showed an altered expression profile, with a relative increase in *mtd1* expression already during the polarity-establishment stage and another induction at first branching. Thus, reduced *mpd1* expression did not result in a simple constitutive increase in *mtd1*, but rather in a stage-dependent redistribution of *mtd1* expression during germination. This altered expression pattern is consistent with a compensatory transcriptional response that may contribute to the maintenance of mannitol accumulation in the *mpd1*-knockdown background. In all isolates, conidia underwent comparable transitions from polarized growth to germ tube emergence and subsequent branching, indicating that reduced transcription of *mpd1as* did not markedly alter the overall germination dynamics under the tested conditions (**Figure 8**).

## Discussion

Conidial germination marks the transition from stress-tolerant dormancy to active growth, requiring spores to rapidly mobilize internal reserves and re-establish metabolic activity. Polyols and soluble sugars are central to this process, providing carbon and energy while supporting osmotic balance, redox homeostasis, and stress protection (Jennings, 1985; Davis et al., 2000; Ruijter et al., 2003; Baltussen et al., 2020). Although mannitol and trehalose are major fungal spore carbohydrates, the regulatory architecture governing their dynamics during germination remains incompletely understood. In particular, it is unclear whether mannitol turnover is governed mainly by a small number of defined enzymatic steps, or whether additional, partially redundant metabolic routes allow fungi to compensate for perturbation of individual pathway components, given diverse spore germination ecologies would be expected for filamentous fungi with various life styles.

Here we have examined the dynamics of soluble storage carbohydrates during conidial germination in *T. asperelloides*, comparing these dynamics with representative Sordariomycetes occupying distinct ecological niches. Our results show that germination is accompanied by pronounced, stage-specific remodeling of carbohydrate pools. In *T. asperelloides*, dormant conidia were enriched in mannitol, which declined sharply during polarity establishment and partially reaccumulated during later germination stages. The early decline in mannitol is consistent with mobilization of stored carbon during germination initiation, whereas its partial recovery at later stages may reflect renewed allocation of carbon toward storage, redox-related functions, or sustained hyphal growth (Dulermo et al., 2009). Trehalose showed an opposing trend, increasing during germ-tube formation and first branching, suggesting that *T. asperelloides* rebuilds a protective carbohydrate pool as growth resumes. Together, these reciprocal dynamics indicate that mannitol and trehalose are not static reserves, but developmentally regulated carbohydrate pools that are differentially mobilized and replenished during germination. The interpretation of these carbohydrate dynamics is supported by the use of quantitative GC-MS profiling with internal standards, which helped reduce analytical variation associated with sugar derivatization and matrix effects.

Comparative sugar profiling across the Sordariomycetes further indicated that storage-carbohydrate composition is not uniform among these fungi. The genetics and physiology of fungi with different lifestyles are expected to be reflected at both the transcriptomic and metabolomic levels, particularly in pathways associated with nutrient acquisition, stress tolerance, host interaction, and developmental transitions (Li et al., 2022; Wang et al., 2023). Recent comparative modeling of fungal sugar metabolism further supports this view (Li et al., 2026), and the genome-scale sugar metabolic model of *N. crassa* differs from that of *A. niger* in pathway redundancy and in the transcriptomic behavior of orthologous sugar-metabolism genes, emphasizing that sugar-metabolic architecture can diverge among filamentous fungi. In the present study, the observed differences in sugar composition among *T. asperelloides*, *M. anisopliae*, *C. militaris*, *F. graminearum*, and *N. crassa* suggest that the balance between mannitol, trehalose, glucose, and other soluble carbohydrates may reflect species-specific physiological strategies rather than a single conserved germination program. This specificity is especially relevant for *T. asperelloides* T203, a mycoparasitic and plant-associated fungus whose interactions with fungal hosts such as *Rhizoctonia solani* have been well documented (Elad et al., 1983; Sivan and Chet, 1989; Viterbo et al., 2002; Viterbo and Horwitz, 2010; Mukherjee et al., 2012). In an ongoing study focusing on pathogen-host interaction between *T. asperelloides* and its potential host *R. solani*, most genes included in the mannitol-associated network retained their typical germination-associated expression patterns when *T. asperelloides* germinated in the presence of *R. solani* (Yakubovich et al., unpublished). This retention suggests that the core metabolic program analyzed here is associated with conidial germination itself. However, this core metabolic program remains relevant to host-associated growth and mycoparasitic interaction. Thus, storage-carbohydrate metabolism in *T. asperelloides* T203 may contribute both to the intrinsic developmental transition from dormancy to active growth and to the physiological readiness required for host-associated development.

A major finding of this study is that reduced expression of *mpd1*, encoding a putative mannitol-1-phosphate dehydrogenase, did not substantially alter mannitol content or the broader soluble-carbohydrate profile during conidial germination. Given the predicted role of MPD in the conversion of fructose-6-phosphate to mannitol-1-phosphate, this result suggests that *mpd1* transcript reduction alone is insufficient to disrupt mannitol homeostasis in *T. asperelloides* under the conditions tested. This lack of a pronounced metabolic phenotype may be explained by adequacy of residual MPD activity in the antisense strains (Kacser and Burns, 1973, 1981; Fell, 1992) by additional enzymes contributing to mannitol biosynthesis or turnover (Vélëz et al., 2007; Krahulec et al., 2011), or by broader flexibility in the connection between mannitol metabolism and central carbon metabolism (Patel and Williamson, 2016). Such buffering is not unusual in central metabolism, where duplicated enzymes, partially overlapping substrate specificities, and alternative pathway branches can reduce the phenotypic impact of perturbing a single enzymatic step (Papp et al., 2004; Li et al., 2022). This interpretation is consistent with studies challenging the classical view of mannitol metabolism as a simple closed cycle (Solomon et al., 2007). In *B. cinerea*, mannitol synthesis and utilization persist even when canonical MPD- and MTDH-associated branches are disrupted, implying the presence of additional metabolic routes (Dulermo et al., 2010). Even double Δ*mpd1* Δ*mtdh* mutants retain substantial mannitol pools and can grow on mannitol as the sole carbon source, strongly arguing for additional mannitol dehydrogenase-like activities, mannitol phosphorylation routes, or parallel pathways feeding into central carbon metabolism. The absence of a strong metabolic phenotype in the *T. asperelloides mpd1* antisense strains is therefore consistent with a model in which mannitol metabolism is buffered by partially redundant or alternative metabolic routes rather than being dependent on a single MPD-catalyzed step (Vélëz et al., 2007; Dulermo et al., 2010).

Bayesian network analysis provided an additional framework to assess this apparent robustness. In the *T. asperelloides* network of selected sugar-metabolism genes, *mpd1/man1* and *mtd1* were associated not only with each other, but also with genes linked to glycolysis and the TCA cycle. This broader connectivity supports the idea that mannitol metabolism during germination is embedded within a central-carbon metabolic module, rather than functioning as an isolated pathway controlled by a single enzymatic step (Dulermo et al., 2010). The inferred links between mannitol-associated genes and downstream metabolic genes are biologically consistent with the mobilization of stored carbohydrates into glycolytic and respiratory metabolism during the transition from dormant conidia to active growth (Ruijter et al., 2003; Dulermo et al., 2010; Li et al., 2022). Because Bayesian-network edges represent inferred associations via limited sampling points rather than experimentally validated causal interactions, these links should be interpreted as hypothesis-generating for further experimental designs. In addition to the transcriptomic considerations which should be taken into account, direct flux measurements should be incorporated to distinguish between *de novo* synthesis and redistribution of existing carbohydrate pools (Longacre et al., 1997; Niklas et al., 2010). Nevertheless, together with the lack of a strong metabolic phenotype in the *mpd1* antisense strains, the network structure supports a model in which mannitol turnover is buffered through coordinated interactions with broader carbon-metabolic pathways.

Comparative Bayesian-network analysis across the Sordariomycetes suggested that the mannitol-associated module contains both conserved and lineage- or species-specific components. Several interactions were shared among sampled species, indicating partial maintenance of a core metabolic architecture, whereas species-specific edges pointed to rewiring of the module in different fungal backgrounds. Notably, shared edges between *T. asperelloides* and *M. anisopliae* involved mannitol-associated genes, consistent with the possibility that network architecture may carry a signal related to conidial storage-carbohydrate profiles. JSD-based comparison of Bayesian-network structural matrices showed partial correspondence with organismal phylogeny, indicating that the inferred networks retain some phylogenetic signal, but also revealed grouping patterns that may reflect differences in sugar-metabolism dynamics. These results suggest that the evolution of storage-carbohydrate metabolism may involve both inherited pathway architecture and ecological or physiological rewiring (Avonce et al., 2006; Askew et al., 2009).

At the transcriptomic level, the predominance of downregulated metabolic-process genes during the initial transition from dormant conidia to polar growth likely reflects extensive remodeling of the dormant-conidial transcriptome, possibly through degradation or dilution of stored transcripts that are no longer required upon germination initiation (van Leeuwen et al., 2013). In contrast, later transitions showed a relatively greater contribution of upregulated genes, consistent with renewed metabolic activation as conidia progressed through germ-tube formation and first branching (Wang et al., 2019). This transition from broad early transcript reduction to later pathway induction is consistent with the observed metabolite dynamics, in which stored carbohydrate pools are first mobilized and later partially rebuilt or redirected into active metabolism.

The Bayesian networks were inferred from a limited number of developmental sampling points and without experimental perturbations. Accordingly, individual edges should be interpreted as candidate associations rather than validated causal interactions (Friedman et al., 2000). Thus, our conclusions were limited to the comparative organization of the mannitol-associated regulatory module across Sordariomycetes without comment on the directionality or hierarchy of genetic interaction. Resolution of the relative contribution of the proposed compensatory mechanisms likely will require complete *mpd1* and *mtd1* knockout combinations, targeted disruption of candidate auxiliary mannitol-pathway genes such as putative mannitol kinases or MDR-type dehydrogenases, and direct metabolic-flux analysis.

Collectively, our metabolite, transcriptomic, and functional analyses support a model in which storage-carbohydrate metabolism is dynamically altered throughout *T. asperelloides* conidial germination rather than being governed by a single dominant metabolic pathway. Dormant conidia are characterized by high mannitol content together with elevated *mpd1* expression, consistent with mannitol functioning as the principal storage carbohydrate during dormancy. Upon germination, both mannitol abundance and *mpd1* transcript levels decline sharply, while trehalose progressively accumulates to become the predominant soluble carbohydrate during germ tube formation. As development proceeds toward first branching, mannitol levels partially recover, coinciding with renewed *mtd1* expression. Importantly, reduction of *mpd1* transcript abundance did not measurably alter mannitol accumulation or germination, indicating that maintenance of storage-carbohydrate homeostasis is robust to perturbation of this pathway. Together with the Bayesian-network analysis, these findings suggest that developmental transitions are accompanied by coordinated redistribution of carbon between mannitol and trehalose through a flexible and partially redundant metabolic network linking storage-carbohydrate metabolism with central carbon metabolism, thereby preserving metabolic and developmental homeostasis during germination. These findings highlight how evolutionary tuning of storage-carbohydrate modules can couple stress protection, reserve mobilization, and developmental progression, and they provide a foundation for future mechanistic work on mannitol flux, redundancy, and ecological adaptation in filamentous fungi.

## Data Availability

The datasets presented in this study can be found in online repositories. The names of the repository/repositories and accession number(s) can be found below: https://www.ncbi.nlm.nih.gov/, GSE207066; https://www.ncbi.nlm.nih.gov/, GSE41484; https://www.ncbi.nlm.nih.gov/, GSE101412; https://www.ncbi.nlm.nih.gov/, GSE168995; https://www.ncbi.nlm.nih.gov/, GSE277787; https://www.ncbi.nlm.nih.gov/, GSE277627.

## Supplementary material

Supplementary Figure 1 A representative GC-MS/MS chromatogram of the analyzed sugars and MRM parameters.

Supplementary Figure 2 Verification of *mpd1* antisense transformants by genomic PCR using the primer pair pLY2_F_1-F (left) and pLY2_F_2-R (right; see **Figure 7A**). The primers flank the *PtrpC–mpd1* antisense–*amdS* cassette and were expected to generate an approximately 6.7-kb amplicon from cassette-containing transformants. No corresponding amplification product was expected from wild-type genomic DNA.

Supplementary Figure 3 Gene expression heatmaps for *T. asperelloides* mannitol expression Bayesian network homologues, based on transcriptomic data previously reported by Wang et al. (2022); Wang et al. (2025); Moonjely and Trail (2024).

Supplementary Figure 4 Sugar and polyol composition during conidial germination in WT and *mpd1RNAas* isolates. Bar plots showing the abundance of selected soluble sugars and polyols in dormant conidia (S1), polar growth (S2), germinated conidia (S3), and first branching (S4) of the wild type and independent *mpd1RNAas* isolates. Overall, all isolates displayed a broadly similar developmental trend, characterized by high mannitol levels in dormant conidia, a marked reduction during early germination, and partial recovery at later stages. Trehalose showed the opposite general pattern, increasing progressively during germination and reaching its highest levels at first branching. Glucose remained relatively abundant throughout germination, whereas fructose, myo-inositol, and ribose were detected at lower levels. These data indicate that the major stage-dependent changes in soluble carbohydrate composition are largely conserved among the analyzed isolates.

Supplementary File 1 Amino acid sequences of RBP1 and RBP2 of the different species used for tree construction.
