# Supplementary material for: Conserved storage-carbohydrate metabolic modules are rewired during germination of Trichoderma asperelloides and other Sordariomycetes
Source: Front Fungal Biol. 2026 Aug 26;7:1930613. doi: 10.3389/ffunb.2026.1930613 (PMC13553963; doi:10.3389/ffunb.2026.1930613)
Supplement: Supplementary file 1 [file SupplementaryFile1.docx]

**Supplementary File 1 — NEXUS Protein Alignment (RPB)**

```
#NEXUS


BEGIN DATA;
 DIMENSIONS NTAX=5 NCHAR=3167;
 FORMAT DATATYPE=PROTEIN INTERLEAVE MISSING=-;

MATRIX
Ncrassa_RPB          MAAYAKDGSQSYGGYGDTYD NDDNYE-NLSTFDDEDDGSD PNAITPEDSWEVISSYFDLK GLVSQQIDSFNEFTTDTVQS LIDEYADLTLDHPSPGDDEG
Fgraminearum_RPB     MD--------------DSYE NDSDYDYGY------DEG-- ---ITPEDCWTVISSFFETK GLASQQTDSFDEFTQRTVQD LVNEYADISLDQQNPPSSYG
Tasperellioides_RPB  MA--------------D-YE DDYDYE-NY---GDEDEG-- ---ITPEDCWTVISSFFETK GLVSQQTDSFDEFTQTTIQD LVNEYSTITLDQPNPPSPPG
Metarhizium_anisopli MA--------------D-YE EDYDYL-TYD--GDDDEG-- ---ITPEDCWTVISSFFDTK GLVSQQTDSFDEFTQTTIQD LVNEYSTITLDQPNPPSAAG
Cordyceps_militarisR MA--------------D-YE DEYDYE-NY----EEDSG-- ---ITPEDCWTVISSFFESK GLVSQQTASFDEFTQTTIQD LVSEYSKISLDQPNPPSTDE

Ncrassa_RPB          REIILRRYDVHFGEIIISRP TLTEATGETISLLPYECRDR NLTYSAPIYCKVTKRARIAV SEKKPLNQLTDDEHDHMART GEHPTALRWIEEESAPPEPP
Fgraminearum_RPB     VPIALRRYEIKFGSVMVSRP SISETDGTVASLLPYECRDR NLTYSSPAYIKITKKVSVAY DKEIPLHEMDEEQQAEYKRT GEHPTKLHWEVEEDGDSM--
Tasperellioides_RPB  RTIALRRYEIKFGSVMVSRP TISETDGTVTSLLPYECRDR NLTYASPLYIKITKKLSAAV EKEIPLHEMDDAQQEEYART GEAPTKLEWEEEEAGEDD--
Metarhizium_anisopli EKVALRRYEIKFGTVMVSRP TISETDGTVTSLLPYECRDR NLTYASPLYINITKKVSRAV EQDIPLKELDDAQREELKRT GEMPTKLVWELEESAEDD--
Cordyceps_militarisR RKISVRRYEIEFGSIMVSRP SISETDGTVTSLLPYECRDR NLTYAAPVYIKITKKVQAAV ETEIPLHELDDAQHTEYAKS GELPTRLAWESEDVSEPE--

Ncrassa_RPB          KDDKINPENLKGYLFLGKIP IMVKSKICHLYMEDDESLFT LNECPYDQGGYFIINGSEKV LIAQERSAANIVQVFKKPPG GSVSYQAEIRSALEKGSRLI
Fgraminearum_RPB     --KEGQP----NMIFVGKMP IMVKSKICHLSQHTDEELFT LNECPYDQGGYFVINGSEKV LIAQERSAANIVQVFKKAQP SPFTYTAEIRSALEKGSRLI
Tasperellioides_RPB  -HNIGKSEDWKDMVFVGKLP IMVKSKICHLSRETDDSLFL VNECPYDQGGYFVINGSEKV LIAQERSAANIVQVFKKAQP SAYTYTAEIRSALEKGSRLI
Metarhizium_anisopli --DTGKSEDWKNMVFVGKLP IMVKSKICHLSREQEDSLFM VNECPYDQGGYFVINGSEKV LIAQERSAANIVQVFKKAQP SPYTYTAEIRSALEKGSRLI
Cordyceps_militarisR --NANKPDAWKDMVFVGKMP VMVKSKVCHLSREHDENLFL VNECPYDQGGYFVINGSEKV LIAQERSAANIVQVFKKAQP SPYTYTAEIRSALEKGSRLI

Ncrassa_RPB          SSLQMRLHTKADQNKGRLAN TVSVTLPYVKEEVSLAIVFR ALGVVSDEDILNHICYDRRD SQMLEALRPCIEEAFCIQDR EVALDYIGKRGN-GSTGINR
Fgraminearum_RPB     SSLMMKILGKGDSARGGFGQ TIQTSLPFVKSDLPVAIVFR ALGVVSDEDILNHICYDRND SQMLEMLRPCIEEAFCVQDR EVALDFIGKRGNRDQASLGR
Tasperellioides_RPB  SSMMLKLYGKGDSARGGFGQ TIHTTLPFVKSDLPVAIVFR ALGVVSDEDILNHICYDRND SQMLEMLRPCIEEAFCVQDR EVALDFIGKRGNRDQAGLGR
Metarhizium_anisopli SSLSLKLYGKGDSARGGFGQ TIHTTLPFVKSDLPIAIVFR ALGVVSDEDILNHICYDRKD SQMLEMLRPCIEEAFCVQDR EVALDFIGKRGNRDQAGLGR
Cordyceps_militarisR SSMMLKLYGKGESARGGFGQ TIHTTLPFVKSDLPVAIVFR ALGVVSDEDILNHICYDRKD SQMLEMLRPCIEEAFCVQDR EVALDFIGKRGNRDQAGLGR

Ncrassa_RPB          NGRIKAAKEMLQKELLPHIS QAEGCETRKAFFLGYMVHKL LQCVLGRRDTDDRDHFGKKR LDLAGPLLAKLFRGIVRRLT QDLMGYMKRCVDTNKHFSLA
Fgraminearum_RPB     EKRVRVAKDILQKETLPHIS QSEGSETRKAFFLGYMVHKL LQCALGRREPDDRDHFGKKR LDLAGPLLAKLFRGIIRRMN QELSNYLKRCVESNRNFNLN
Tasperellioides_RPB  EKRVRVAKDILQKETLPHIS QTEGSETRKAFFLGYMVHKL LQCALGRREPDDRDHFGKKR LDLAGPLLAKLFRGIMRRMN TELANYLRRCVEGNRHFNLA
Metarhizium_anisopli DKRVRVAKDILQKETLPHIS QTEGSETRKAFFLGYMVHKL LQCALGRREPDDRDHFGKKR LDLAGPLLAKLFRGIIRRMN TELSNYLRRCVEGNRHFNLA
Cordyceps_militarisR DKRIRVARDILQKETLPHIS QEAGSETRKAFFIGYMVHKL LQCALGRREPDDRDHFGKKR LDLAGPLLAKLFRGIMRRMH SELANYLRRCVEGNRHFNLA

Ncrassa_RPB          LGIKASTLTNGLKYSLATGN WGDQKKAMSSTAGVSQVLNR YTFSSTLSHLRRTNTPIGRD GKLAKPRQLHNTHWGLVCPA ETPEGQACGLVKNLSLMCFV
Fgraminearum_RPB     VAIKPGTLSNGLKYSLATGN WGDQKKAASSTAGVSQVLNR YTFASTLSHLRRTNTPIGRD GKLAKPRQLHNTHWGLVCPA ETPEGQACGLVKNLSLMCYV
Tasperellioides_RPB  VGIKPGTLSNGLKYSLATGN WGDQKKAMSSTAGVSQVLNR YTFASTLSHLRRTNTPIGRD GKLAKPRQLHNTHWGLVCPA ETPEGQACGLVKNLSLMCYV
Metarhizium_anisopli VGIKPGTLSNGLKYSLATGN WGDQKKAMSSTAGVSQVLNR YTFASTLSHLRRTNTPIGRD GKLAKPRQLHNTHWGLVCPA ETPEGQACGLVKNLSLMCYV
Cordyceps_militarisR VGIKPGTLSNGLKYSLATGN WGDQKKAANSTAGVSQVLNR YTFASTLSHLRRTNTPIGRD GKLAKPRQLHNTHWGLVCPA ETPEGQACGLVKNLSLMCYV

Ncrassa_RPB          SVGTLADPIIEFMIARNMEV LEEYEPLRYPNATKVFVNGT WVGVHQDPKHLVTLVQNLRR KNIISFEVSLVRDIRDREFK IFSDAGRVMRPLFVVEQEDN
Fgraminearum_RPB     SVGSPGEPLIDFMVSRGMEV VEEYEPTRYPHATKVFVNGS WVGVHPEPRALVNSVLETRR KSYLQFEVSLVRDIRDREFK IFSDAGRVMRPVFTVQQEDD
Tasperellioides_RPB  SVGSPSEPLIEFMINRGMEV VEEYEPLRYPHATKIFVNGV WVGIHQDPKHLVQQVVDTRR KSYLQYEVSLVREIRDQEFK IFSDAGRVMRPVFTVQQDEE
Metarhizium_anisopli SVGSPAEPLIEFMINRGMEV VEEYEPLRYPHATKIFVNGV WVGVHQDPKHLVSQVLDTRR KSYLQYEVSLVREIRDQEFK IFSDAGRVMRPVFTVQQEDD
Cordyceps_militarisR SVGSPAEPLIDFMINRGMEV IEEYEPLRYPHATKIFVNGT WVGVHQDPKHLVDQVFDTRR KSYLQYEVSLIRDIRDQEFK IFSDAGRVMRPVFTVQQKND

Ncrassa_RPB          SESGVEKGQLILTKEHIRRL EQDK-ELGKYHEDYFGWQGL LESGAIEYLDAEEEETAMIC MTPEDLDTYRQHKF------ -----KGREFEDD-AANRGN
Fgraminearum_RPB     YETGLTKGQLVLTKDLVNKI AQEQAEPPSDPSERIGWQGL IRSGAVEYIDAEEEETAMIC MTPEDLEIYREQKRDEVNLT EEEKQAKLEAEKREQEEERN
Tasperellioides_RPB  SDTGIPKGHLVLTKDLVNKL AQEQAEPPEDPSMKIGWEGL IRAGAVEYLDAEEEETAMIC MTPEDLELYRAQK------- -----AGIATEED-VGDDPN
Metarhizium_anisopli PETGIEKGHLVLTKELVNKL AKEQAEPPEDPSEKIGWEGL IRAGAVEYLDAEEEETSMIC MTPEDLELYRLQK------- -----AGVALDDD-IGDDLN
Cordyceps_militarisR PETGLEKGQLGLTKDLVNKL AQEQADPPDDSELKMGWEGL IKAGAIEYLDAEEEETSMIC MTPEDLELYRLQK------- -----AGLSVDDDHEGGDLN

Ncrassa_RPB          SRIKTKFNPTTHMYTHCEIH PSMLLGICASIIPFPDHNQS PRNTYQSAMGKQAMGFFLTN YSRRMDTMANILYYPQKPLA TTRSMEYLKFRELPAGQNAI
Fgraminearum_RPB     KRLKTKVNPTTHMYTHCEIH PSMILGICASIIPFPDHNQS PRNTYQSAMGKQAMGFFLTN YSRRMDTMANILYYPQKPLA TTRSMEFLKFRELPAGQNAI
Tasperellioides_RPB  KRLKTRTNPTTHMYTHCEIH PSMILGICASIIPFPDHNQS PRNTYQSAMGKQAMGFFLTN YSRRMDTMANILYYPQKPLG TTRSMEFLKFRELPAGQNAI
Metarhizium_anisopli KRLKTKTNPTTHMYTHCEIH PSMILGICASIIPFPDHNQS PRNTYQSAMGKQAMGFFLTN YSRRMDTMANILYYPQKPLA TTRSMEFLKFRELPAGQNAI
Cordyceps_militarisR KRLKTKTNPTTHMYTHCEIH PSMILGICASIIPFPDHNQS PRNTYQSAMGKQAMGFFLTN YSRRMDTMANILYYPQKPLA TTRSMEFLKFRELPAGQNAI

Ncrassa_RPB          VAIACYSGYNQEDSVIMNQS SIDRGIFRSLFFRSYTDCEK RVGINIVEQFEKPDRSNTLR PKHGTYDKLENDGIIAPGIR VTGDDIIIGKTSPINPDNQE
Fgraminearum_RPB     VAIACYSGYNQEDSVIMNQS SIDRGLFRSLFFRSYSDQEK KVGLNYTEIFEKPFQQTTLR MKHGTYDKLDEDGIVAPGVR VSGEDIIIGKTAPIDQENQD
Tasperellioides_RPB  VAIACYSGYNQEDSVIMNQS SIDRGLFRSLFFRSYSDQEK KVGLNYTEVFEKPFHQNTLR MKHGTYDKLDEDGIVAPGVR VSGEDIIIGKTAPIDADTQD
Metarhizium_anisopli VAIACYSGYNQEDSVIMNQS SIDRGLFRSLFFRSYSDQEK KVGLNYTEVFEKPFHQSTLR MKHGTYDKLDDDGIVAPGVR VSGEDIIIGKTAPIDQESQD
Cordyceps_militarisR VAIACYSGYNQEDSVIMNQS SIDRGLFRSLFFRSYSDQEK KVGLNYTEIFEKPFHQSTLR MKHGTYDKLDEDGIVAPGVR VSGEDIIIGKTAPIDPETQD

Ncrassa_RPB          LGQRTAQHVKRDASTPLRST ESGIVDSVAFTTNQDGLRYV KVRVRTTKIPQIGDKFASRH GQKGTIGVTYRQEDMPFTAE GITPDIIINPHAIPSRMTIA
Fgraminearum_RPB     LGTRTQTHQRRDISTPLRST ENGIVDQVILTVNADNVKYV KVRVRTTKIPQIGDKFASRH GQKGTIGVTYRQEDMPFSRE GLTPDIIINPHAIPSRMTIA
Tasperellioides_RPB  LGTRTTMHQRRDISTPLRST ENGIVDSVIVTVNADNVKYV KVRVRTTKIPQIGDKFASRH GQKGTIGVTYRQEDMPFTRE GITPDIIINPHAIPSRMTIA
Metarhizium_anisopli LGTRTSVHQRRDISTPLRST ENGIVDSVILTVNADNVKYV KVRVRTTKIPQIGDKFASRH GQKGTIGVTYRQEDMPFTRE GVTPDIIINPHAIPSRMTIA
Cordyceps_militarisR LGTRTTAHQRRDISTPLRST ENGIVDQVIVTVNADNVKYV KVRVRTTKIPQIGDKFASRH GQKGTIGVTYRQEDMPFTRE GVTPDIIINPHAIPSRMTIA

Ncrassa_RPB          HLIECLLSKVGALKGMEGDA TPFTDVTVDSVSNLLREHGY QSRGFEIMYHGHTGKKLRAQ VFFGPTYYQRLRHMVDDKIH ARARGPVQIMTRQPVEGRAR
Fgraminearum_RPB     HLIECLLSKVSTLEGMEGDA TPFTDVTVDSVSDILRKHGY QSRGFEVMYNGHTGRKLRAQ VFFGPTYYQRLRHMVDDKIH ARARGPVQIMTRQPVEGRAR
Tasperellioides_RPB  HLIECLLSKVSTLEGMEGDA TPFTDVTVDSVSELLRKHGY QSRGFEIMYNGHTGRKLRAQ VFFGPTYYQRLRHMVDDKIH ARARGPVQIMTRQPVEGRAR
Metarhizium_anisopli HLIECLLSKVSTLEGMEGDA TPFTDVTVDSVSELLRKHGY QSRGFEIMYNGHTGKKLRAQ VFFGPTYYQRLRHMVDDKIH ARARGPVQIMTRQPVEGRAR
Cordyceps_militarisR HLIECLLSKVSTLEGMEGDA TPFTDVTVDSVSDLLRKHGY QSRGFEIMYNGHTGKKLRAQ VFFGPTYYQRLRHMVDDKIH ARARGPVQIMTRQPVEGRAR

Ncrassa_RPB          DGGLRFGEMERDCMIAHGAA AFLKERLFEVSDAFRVHICE ICGLMTPIANLTKQSFECRP CKNKTKIAQVHMPYAAKLLF QELMSMNIASRLFTSRSGIS
Fgraminearum_RPB     DGGLRFGEMERDCMIAHGAA AFLKERLFEVSDAFRVHVCE ICGLMTPIANLSKQSFECRP CKNKTKIAQIHIPYAAKLLF QELQAMNIAARMFTNRSGVS
Tasperellioides_RPB  DGGLRFGEMERDCMIAHGAA SFLKERLFEVSDAFRVHICE ICGLMTPIANLSKQSFECRP CKNKTKIAQIHIPYAAKLLF QELQSMNIAARMFTDRSGAS
Metarhizium_anisopli DGGLRFGEMERDCMIAHGAA AFLKERLFEVSDAFRVHICE ICGLMTPIANLSKQSFECRP CKNKTKIAQIHIPYAAKLLF QELQSMNIAARMFTNRSGAS
Cordyceps_militarisR DGGLRFGEMERDCMIAHGAA AFLKERLFEVSDAFRVHICE ICGLMTPIANLSKQSFECRP CKNKTKIAQIHIPYAAKLLF QELQSMNIAARMFTDRSGAS

Ncrassa_RPB          VR------------RPRPRP RPRPRPRPRPRPRPRPRPRP RPRPRPRPRPRPRPRPRPRP RPRPRPRPRPRPRPRPRPMA NLYFAHSSAPLRTIKEIQFG
Fgraminearum_RPB     IR------------RPRPRP RPRPRPRPRPRPRPRPRPRP RPRPRPRPRPRPRPRPRPRP RPRPRPRPRPRPRPRPRPMA NLYFTHSSAPLKTVEEIQFG
Tasperellioides_RPB  VR------------RPRPRP RPRPRPRPRPRPRPRPRPRP RPRPRPRPRPRPRPRPRPRP RPRPRPRPRPRPRRPRPPMA NLYFTHSSAPLKTVQEIQFG
Metarhizium_anisopli IR------------RPRPRP RPRPRPRPRPRPRPRPRPRP RPRPRPRPRPRPRPRPRPRP RPRPRPRPRPRPRPRPRPMA NYHPPFSSAPLKTVEEIQFG
Cordyceps_militarisR IR------------RPRPRP RPRPRPRPRPRPRPRPRPRP RPRPRPRPRPRPRPRPRPRP RPRPRPRPRPRPRPRPRPMA NLPFPHSSAPLKTVEEIQFG

Ncrassa_RPB          LLSPEEIKGMSVAHIVYPET MDETRTKPRDGGLNDPLLGS VDRQFKCKTCGENMSECPGH FGHIELARPVYHPGFIKRVK KMLEIVCHNCSKVLADRNDK
Fgraminearum_RPB     LMSPEEIKNMSVCHIVYPET MDENKTKPRDGGLNDLLLGS IDRSFRCKTCTQAMGECPGH FGHIELAKPVYHPGFIKKVK KILEIVCHNCSKVLADSRDP
Tasperellioides_RPB  LMSPEEIKSMSVAHILYPET MEEGGTKPRDGGLNDPLLGS IDRQFKCKTCTQAMGECPGH FGHIELAKPVYHPGFIKKVK KILEIVCHNCSKVLADESDP
Metarhizium_anisopli LMSPEEIKNMSVCHILYPET MDESRTKPRDGGLNDPLLGS IDRGFKCKTCTQSMSDCPGH FGHIELAKPVYHPGFIKKVK KILEIVCHNCSKVLADTSDP
Cordyceps_militarisR MLSPEEIKNMSVAHILYPET MEENKTTPRDGGLNDPLLGS IDRQFKCKTCAQPMSECPGH FGHIELAKPVYHPGFIKKVK KVLEIVCHNCSKVLADESDP

Ncrassa_RPB          EFAAAMRIRDPKVRFNRVWA VCKKKRRCENEVVKKSD-DD EFNPLADAKGELPGHGGCGN DHPTVRQQALTLYAQFDTK- DEEG--VKSKEKKIITPEMA
Fgraminearum_RPB     EFAAAINTRDPKVRFHRVWE ACKKKKRCENDEPKQK--DD EFAATLKTG-PLEGHGGCGN VQPAVRQGALQLKAAFEVTV DEDGQKTKKKETIPITPETA
Tasperellioides_RPB  EFVAAINTRDAKLRFKRVWA VCKKKRRCENDDRSEKNKDE EFAPGMKPA-VVESHGGCGN VQPQVRQVALQLKAAFEVV- QEDG--PKRKETAPITPEMA
Metarhizium_anisopli EFVTAINTRDAKLRFNRVWA VCKKKRRCENEDRNEKN-DD EFAPGMKPV--AHNHGGCGN VQPQVRQAALQLKAAFDVA- QEDG--PKRRETVPITPEMA
Cordyceps_militarisR EFVAAVRTRDSKVRFKRIWA VCKKKRKCENEERQDKNKDE EFAPGVKNT-VLEGHGGCGN MQPQVRQAALQLKAAFEVN- SEEG--PKRKETVNISAEMA

Ncrassa_RPB          LNILHNMTESDMIDIGINIS QARPEWMIITVLPVPPPPVR PSISMDGTGTGMRNEDDLTY KLGDIIRANGNVRQAIQEGS PAHIINDFENLLQYHVATYM
Fgraminearum_RPB     HGILRRISEEDLVNMGLNSD YARPEWMVLTVLPVPPPPVR PSISMDGTGTGMRNEDDLTY KLGDIIRANGNVKQAIREGS PQHIARDFEELLQYHVATYM
Tasperellioides_RPB  HSILRRISEKDLRNMGLNSD YARPEWMIITVLPVPPPPVR PSISMDGTGTGMRNEDDLTY KLGDIIRANGNVKQAIREGS PQHIARDFEELLQYHVATYM
Metarhizium_anisopli HGILRRISEEDLRHMGLNSD YARPEWMILTVLPVPPPPVR PSISMDGTGTGMRNEDDLTY KLGDIIRANGNVKQAIREGS PQHIARDFEELLQYHVATYM
Cordyceps_militarisR HGILRRISERDLHNMGLNSD YARPEWMIITVLPVPPPPVR PSISMDGTGTGTRNEDDLTY KLGDIIRANGNVKQAIREGS PQHIARDFEELLQYHVATYM

Ncrassa_RPB          DNDIAGQPQALQKSGRPVKA IRARLKGKEGRLRGNLMGKR VDFSARTVITGDANISLDEV GVPRSIARTLTYPETVTPYN IGKLTQYVQNGPNEHPGAKY
Fgraminearum_RPB     DNDIAGQPRALQKSGRPVKA IRARLKGKEGRLRGNLMGKR VDFSARTVITGDANLSLHEV GVPRSIARTLTYPETVTPYN IEKLHQLVENGPNEHPGAQY
Tasperellioides_RPB  DNDIAGQPRALQKSGRPVKA IRARLKGKEGRLRGNLMGKR VDFSARTVITGDANLSLHEV GVPRSIARTLTYPETVTPYN IGKLHQLVENGPNEHPGAKY
Metarhizium_anisopli DNDIAGQPRALQKSGRPVKA IRARLKGKEGRLRGNLMGKR VDFSARTVITGDANLSLHEV GVPRSIARTLTYPETVTPYN IGKLHQLVENGPNEHPGAKY
Cordyceps_militarisR DNDIAGQPRALQKSGRPVKA IRARLKGKEGRLRGNLMGKR VDFSARTVITGDANLSLHEV GVPRSIARTLTYPETVTPYN IAKLHQLVENGPNEHPGAKY

Ncrassa_RPB          VIRSDGSRVDLRHQRRTGPL QLEYGWKVERHLIDGDYIIF NRQPSLHKESMMGHRVKVMP YSTFRLNLSVTSPYNADFDG DEMNLHVPQTEETRAEVKEL
Fgraminearum_RPB     VVRPDGSRIDLRHHRRAGAI SLEYGWKVERHLMTGDYIIF NRQPSLHKESMMGHRVRVMP YSTFRLNLSVTSPYNADFDG DEMNLHVPQSEETRAEVKEL
Tasperellioides_RPB  VIRSDGTRIDLRHHRRAAQI SLEYGWKVERHLMDGDYIIF NRQPSLHKESMMGHRVRVMP YSTFRLNLSVTSPYNADFDG DEMNLHVPQSEETRAEVKEL
Metarhizium_anisopli VIRADGTRIDLRHHRRAAQI SLEYGWKVERHLMDGDYIIF NRQPSLHKESMMGHQVRVMP YSTFRLNLSVTSPYNADFDG DEMNLHVPQTEETRAEVKEL
Cordyceps_militarisR VIRSDGTRIDLRHHRRAAQI SLEYGWKVERHLIDGDYIIF NRQPSLHKESMMGHRVKVMP YSTFRLNLSVTSPYNADFDG DEMNLHVPQTEETRAEIKEL

Ncrassa_RPB          CMVPLNIVSPQRNGPLMGIV QDTLAGAYKLCRRDVFLTKE EVMNIMLWVPEWDGIIPVPA ILKPRPRWTGKQIISMVVPK IINLKNIDE----DDGECPL
Fgraminearum_RPB     CLVPLNIVSPQKNGPLMGIV QDSLAGAYKMCRRDVFLTKE QIMNCMLWVPNWDGVIPQPA IYKPRPRWTGKQLISMVIPK EVTIFNGTD----SGESAPL
Tasperellioides_RPB  CLVPNNIVSPQKNGPLMGIV QDSLAGAYKLCRRDTFLDKN QVMNLMLWVPNWDGVIPQPA ILKPRPRWTGKQIISMVIPK EISLHAPED-----KSDSPL
Metarhizium_anisopli CLVPNNIVSPQKNGPLMGIV QDSLAGVYKLCRRDTFIDKE MVMNMMLWVPNWDGVIPQPA ILKPRPRWTGKQLISMVIPQ EISLHAPEG-----DSDIPL
Cordyceps_militarisR CLVPNNIVSPQKNGPLMGIV QDSLAGCYKLCRRDTFIDKE LVQNIMLWVPNWDGVIPQPA ILKPRPRWTGKQIISMVIPA EISLYSKED-----KLDNPK

Ncrassa_RPB          KDKGILIQQGEVIFGLMTKK IVGAASGGIVHLCYNELGPS GAMDFLNGCQRVVNYWLLHN GHSIGIGDTIPDAATIAKVQ THIDDAKAEVKQLTDMATNN
Fgraminearum_RPB     KDEGLLIQAGQLMYGLLTKK SVGASAGGIVHISYNELGPE GAMAFLNGVQQVVTYWLLQN GHSIGIGDTIPDAVTIAKVQ AHIDDEKAEVARLTAMATAN
Tasperellioides_RPB  KDAGLLIQAGELMYGLMKKK NIGSASGGVIHLCYNELGPE GAMAFLNGVQQVVTYWLLQN GHSIGIGDTIPDKQTIEKVQ VHIDTQKAEVARLTAMATNN
Metarhizium_anisopli KDTGLLIQSGELLYGLLKKK NVGAAAGGIIHLCYNELGPD GAMAFLNGVQQVVTYWLLNT GHSIGIGDTVPDKQTIEKIQ VHIDTQKAEVAKLTAQATAN
Cordyceps_militarisR HDAGMLIQSGQLIYGLLKKK YVGAAAGGIIHLCYNELGPD GAMAFLNGVQQVVTYWLLNT GHSIGIGDTIPDKETIDKIQ AHIDEHKAEVARLTAQATAN

Ncrassa_RPB          QLEPLPGMNIRETFENKVSK ALNTARDKAGTTTESSLKDI NNAVTMALSGSKGSSINISQ MTALVGQQIVEGKRIPFGFK YRTLPHFTKDDYSPEARGFV
Fgraminearum_RPB     ELEALPGMNVRATFENKVSM ALNQARDKAGTTTQKSLKDS NNAVTMASSGSKGSSINISQ MTALVGQQIVEGKRIPFGFK YRTLPHFTKDDYSPEARGFV
Tasperellioides_RPB  ELEALPGMNVRATFENKVSM ALNMARDQAGTTTQKSLKDS NNAVTMAESGSKGSSINISQ MTALVGQQIVEGKRIPFGFK YRTLPHFTKDDYSPEARGFV
Metarhizium_anisopli ELEALPGMNVRATFENKVSM ALNSARDQAGTTTQKSLKDS NNAVTMSESGSKGSSINISQ MTALVGQQIVEGKRIPFGFK YRTLPHFTKDDYSPEARGFV
Cordyceps_militarisR ELEALPGMNVRATFESKVSL ALNMARDQAGTSTQKSLKDS NNAVTMSESGSKGSSINISQ MTALVGQQIVEGKRIPFGFK YRTLPHFTKDDYSPEARGFV

Ncrassa_RPB          ENSYLRGLTPSEFFFHAMAG REGLIDTAVKTAETGYIQRR LVKALEDSAAQYDGTVRNSL GDIIQFVYGEDGLDGIAIEN QPVDHFTISNKAFDKRYRLD
Fgraminearum_RPB     ENSYLRGLTPSEFFFHAMAG REGLIDTAVKTAETGYIQRR LVKALEDLSARYDGTVRNSL GDIVQFLYGEDGLDAMIIEN QKLGILNMSNTAFEKKYRLD
Tasperellioides_RPB  ENSYLRGLTPTEFFFHAMAG REGLIDTAVKTAETGYIQRR LVKALEDLNAQYDGTVRNSL GDIIQFLYGEDGLDAMCIEK QKLGILKMSDEAFEKKYRLD
Metarhizium_anisopli ENSYLRGLTPSEFFFHAMAG REGLIDTAVKTAETGYIQRR LVKALEDLSARYDGTVRNSL GDIVQFLYGEDGLDAMCIEK QKLGILKMSDAAFEKKYRLD
Cordyceps_militarisR ENSYLRGLTPTEFFFHAMAG REGLIDTAVKTAETGYIQRR LVKALEDLSARYDGTVRNSL GDVIQFLYGEDGLDAMCIER QRLGTIKMSDAAFERQYRLD

Ncrassa_RPB          VMDEEVSSGELEALEYGKEL ASDPTVQELLDQEYEQLSVD RHMLREIQRTKGNNDTKMQL PLNIIRIIDTAKKLFKVDET ARSDLTPKDVIPAVKAMLDR
Fgraminearum_RPB     LANP--PEWFKHDYEFGNEL TGDRPSMALLDEEWERLVHD RTRIRAINRAKGNEEM-MQL PLNITRIIESAKRVFNVKAN DRSNLRPSDVIPAVQNMLDN
Tasperellioides_RPB  LANP--PDWFRKDYEYGNEL TGDKASMALLDEEWDQLLAD RREVREINRSKMGEEM-MQL PLNIGRMIETAKRVFNVKAN DRSNLRPSDVIPVVRNVINK
Metarhizium_anisopli LANP--PDWFKKDYEYGNEL AGDKESMDLLDSEWETLLSD RQTVRLINKSKMGEEM-MQL PLNIGRMIETAKRVFNVRAT DRSNLRPADVIPRIQNLLSE
Cordyceps_militarisR LANP--PEWFRKDYEYGNEL AGDKASMELLDMEWDTLLAD RRAVRDINKHKMGEEM-MQL PLNIGRIIESAKRVFNIRET DRSNLRPSDVITTVQTLLSG

Ncrassa_RPB          MIVVRGDDPISKEADYNATI LFKAQLRSRLAFKRLAVQDR INKLAFNHILGELENRWSRS MVSPGEMVGVLAAQSIGEPA TQMTLNTFHFAGVSSKNVTL
Fgraminearum_RPB     MKIVRGTDPISLEADANASI LFKGLLRSRLAFKEVVKEHR LNRLAFDHILGELQNRWDRA FVNPGEMVGVLAAQSIGEPA TQMTLNTFHFAGVSSKNVTL
Tasperellioides_RPB  LKIVRGEDQISKEADTSATI LFKALLRSRLAFKEVVKEHR LNKLAFEHVLGELQNRWDRS FVNPGEMVGVLAAQSIGEPA TQMTLNTFHFAGVSSKNVTL
Metarhizium_anisopli LRIVRGSDPISMEADLNATI LFKALVRSRLAFKEIVKVHR LNKLAFDHVLGELQNRWDRS FVSPGEMVGVLAAQSIGEPA TQMTLNTFHFAGVSSKNVTL
Cordyceps_militarisR MKIVRGSDPISLEADFNATI LFKALLRSRLAFKEIVYVHR LNKLAFDHVLGELQNRWDRA FVSPGEMVGVLAAQSIGEPA TQMTLNTFHFAGVSSKNVTL

Ncrassa_RPB          GVPRLKEILNVASNIKTPSM MVYLDSKAATQEEAKKMRSA VEHTSLRSVTAVTEIYYDPD ITSTNIPEDYDLVESYFLIP DQSGDEPDPIENQSRWLLRI
Fgraminearum_RPB     GVPRLKEILNLAKNIKTPSM AVYLNTPLAKQEQAKKLRSM VEYTNLRSVTSVTEIYYDPN VTETNIPEDLDMVESYYMIP D---ESVDPTANQSRWLLRI
Tasperellioides_RPB  GVPRLKEILNLAKNIKTPSM AVYLDTPLAKQEQAKKLRSL VEYTNLRSITSVTEIYYDPD VQSTTIPEDLDMVESYFLIP D---DSQDTIDQQSRWLLRI
Metarhizium_anisopli GVPRLKEILNLAKDIKTPSM AVYLNTKLGTQEQAKKLRSM VEYTNLRSITSVTEIYYDPD IQGTTIAEDMDMVESYFLIP D---DGQDNTDQQSRWLLRI
Cordyceps_militarisR GVPRLKEILNLAKNIKTPSM AVYLDTKLGTQEQAKKLRSL VEYTNLRSVTSVTEIYYDPD VQATNITEDVDMVESYFMIP D---DGQDTIHRQSRWLLRI

Ncrassa_RPB          TLDRQKMLDKGLRVEDVAHR IKEVYKKDVAVVFSDNNAEE MVIRIRVIKADDDKDEDGN- KIIEDDVMLKRLEKHLLDGC TLRGVEGIERAFLNKGVKLI
Fgraminearum_RPB     TLDRQKLLDKEIKIDDVAQR IKEEYPTDLAVIFSDNNADE QVIRIRTIHTG-DKDDDGDG GRMEDDVMLKRLEAHLLDTL TLRGVPGIERAFLTKGTKLS
Tasperellioides_RPB  TLDRQKLLDKEIKIDDVAQR IKEEYPNDLAVIFSDNNADE QVIRIRTIRQD-EKDEDGE- KKIEDDVMLKRLEAHLLDTL TLRGVPGVERAFLTKGTRLV
Metarhizium_anisopli TLDRQKLLDKEIRIDDVAQR IKEEYPNDLAVIFSDNNADE QVIRIRTVRQSDEKDEDGE- KKIEDDVMLKRLEAHLLDTL TLRGVPGIERAFLTKGTRLT
Cordyceps_militarisR TLDRQKLLDKEITIDDVAAC IKEEYATDLAIIFSDNNADE QVIRIRTIRRDDGKDEDPD- TKIEDDVMLKRLEAHLLDTM TLRGVPGVERAFLSKGTRLI

Ncrassa_RPB          EAKDGSMKHSKVDDECQEWY LDTQGTSLREVLTVEGVDTK RTTTNDLWQVVDVFGIEGAR AALMHELTAVLAFDGSYVNH RHLALLCDVMTYRGSIAAVT
Fgraminearum_RPB     EDDDGALLAIKDDPRCTQWY LDTSGTALRDVLAVDGVDAT RTYTNDLYQIVEVFGIEAAR SALAKELTNVLAFDGSYVNH RHIALLVDVMTYRGVISAVT
Tasperellioides_RPB  EAADGSELALKDDPRCTQWY LDTSGSALREVLAVPGVDPT RTYTNDLWQIIEVFGIEGTR AALVKELTAVLAFDGSYVNH RHIALLCDVMTYRGSISAVT
Metarhizium_anisopli VGEDGSELAIKDDPRCTQWY LDTSGSALRDVLAVDGVDAS RTYTNDLWQIVEVFGIEAAR SALVKELTNVLAFDGSYVNH RHIALLVDVMTYRGSISAVT
Cordyceps_militarisR EDEDGSELALIDNPKCTQWF LDTSGSALREVLAVPGVDPR RTYSNDLYQITEVFGVEAVR SALIKELSNVLSFDGSYVNH RHIALLCDIMTYRGVISAVT

Ncrassa_RPB          RHGINRADTGALMRCSFEET VEILLEAAAVGELDDCRGIS ENVMLGQMAPMGTGAFDVFL DPKMLETVISDNSRMGLMPG MTVKGSQLDGAATPYDTGSP
Fgraminearum_RPB     RHGINRADTGALMRCSFEET VEILLEAAATGELDDCRGIS ENVMLGQMAPMGTGNFDVLL DPKMLETVISDNSRMGLMAG MPTKSGDIEGAATPYDTGSP
Tasperellioides_RPB  RHGINRADTGALMRCSFEET VEILLEAAATGELDDCRGIS ENVMLGQMAPMGTGNFDVYL DPKMLETVISDNSRMGLMPG MPVKEGDVEGAATPYDTGSP
Metarhizium_anisopli RHGINRADTGALMRCSFEET VEILLEAAATGELDDCRGIS ENVMLGQLAPMGTGNFDVLL DPKMLETVISDNSRMGLMPG MPVKDGEAEGAATPYDTGSP
Cordyceps_militarisR RHGINRADTGALMRCSFEET VEILLEAAASGELDDCRGIS ENVMLGQLAPMGTGNFDVYL DPKMLETVISDNSRMGLMPG MPAKEGEGEGAATPYDSGSP

Ncrassa_RPB          MA-DSGFLGSYSPTMGNFSP IQGAGSDSPTNNGFGTDYGG VG-GYGSGTSYSSATSPRAT SPF---TPTSPFSY---GGY SPSSPAAGYSPSSPLLDSAG
Fgraminearum_RPB     MA-DSGYLSMSSPAAGNFSP IQGAGSDTPA--GFDTVYGG GSVGFGGTTPMSPYN-RGAA SPFSSTSPTSPFS------Y SPTSPNMGYSPTSPLIDGGG
Tasperellioides_RPB  MG-DSGYLSLNSPAAGNFSP IQGAGSETPA--GFGTEYGG G--GFGGIGSMSPYSMRGAT SPF-STSPTSPFSSGM-GGY SPTSPNAGYSPTSPMIDGGI
Metarhizium_anisopli MSNDSGYMSMNSPAAGNFSP IQGAGSETPA--GFGTEYGG G--GFGNVGSMSPYS-RGAT SPF-STSPTSPFSSGM-VGY SPSSPNAGYSPTSPLLDGGA
Cordyceps_militarisR MG-ESGYLSMASPAAGNFSP IQGAGSETPT--GFNTEYGG ---GFGTNGAMSPYS-RGAS SPF-STSPTSPFSAGM-GGY SPSSPNTGYSPTSPMLDGGA

Ncrassa_RPB          --RFA-SSPQFSPSSPSFSP TSPMLRPGSPTSPSYSPTSP SYSPASPAAAR-YSPTSPAQ QFSPTSPSYSPTSPSYSPAS PAFQA----TSPSYSPASPT
Fgraminearum_RPB     MGRYGPTSPSFSPSSPSFSP TSPMLRPTSPASPSYSPTSP SYSPTSPSSPRHYSPTSPAQ FNSPTSPSYSPASPNYSPTS PNVHGAGGPTSPSYSPASPS
Tasperellioides_RPB  -GRYA-TSPSFSPSSPSFSP TSPMLRPTSPASPNYSPTSP SYSPASPSSPRHYSPTSPAQ FNSPTSPSYSPASPNYSPAS PNLHG-VGATSPSYSPASPS
Metarhizium_anisopli -GRYA-TSPSFSPSSPSFSP TSPMLRPTSPASPNYSPTSP SYSPTSPTSPRHYSPTSPAQ FNSPTSPSYSPASPNYSPAS PNLHGAAGATSPSYSPASPS
Cordyceps_militarisR --RYA-TSPSFSPSSPSFSP TSPMLRPTSPASPNYSPTSP SYSPTSPTSPRHYSPTSPAQ FNSPTSPSYSPASPNYSPAS PNLHG---ATSPSYSPASPS

Ncrassa_RPB          WSPTSPDAYSPTSPSFQRSP GQQLSPTSPTGYSPTSPQYS PRTPGRNNSGGN---GDQYS PTSPSND
Fgraminearum_RPB     WSPTSPEAYSPTSPTFQRSP TTQQSPTSP-SYSPTSPAFS PRTPGPGTS------GNQYS PNSPSND
Tasperellioides_RPB  WSPTSPEAYSPTSPSFSKSP GSQQSPTSP-SYSPTSPSFS PRTPGPGAS------GNQYS PNSPAND
Metarhizium_anisopli WSPTSPEAYSPTSPSFQRSP GNQQSPTSP-SYSPTSPAFS PRTPGPGNS------GNQYS PNSPSND
Cordyceps_militarisR WSPTSPDAYSPTSPSFGRSP GQQQSPTSP-GYSPTSPQFS PRTPGPPGSGGDAGTGNQYS PSSPNNE

;
end;


begin mrbayes;
outgroup Ncrassa_RPB;
set autoclose=yes;
prset aamodelpr=mixed;
mcmcp ngen=1000000 samplefreq=1000 nchains=4 savebrlens=yes filename=BarSugar;
mcmc nruns=1;
sump burnin=1000;
sumt burnin=1000; contype=allcompat;
end;
```
