# Supplementary material for: Conserved storage-carbohydrate metabolic modules are rewired during germination of Trichoderma asperelloides and other Sordariomycetes
Source: Front Fungal Biol. 2026 Aug 26;7:1930613. doi: 10.3389/ffunb.2026.1930613 (PMC13553963; doi:10.3389/ffunb.2026.1930613)
Supplement: Supplementary file 2 [file Table1.docx]

**Table S1**. MRM parameters of the analyzed sugars

| Compound name | Retention time, min | Q1 mass | Q3 mass | Collision energy |
| --- | --- | --- | --- | --- |
| Xylitol | 8.5 | 307 | 217 | 10 |
|  |  | 307 | 103 | 10 |
|  |  | 319 | 229 | 12 |
|  |  | 319 | 157 | 12 |
| Arabinose | 9.16, 9.26 | 307 | 103 | 10 |
|  |  | 320 | 191 | 12 |
|  |  | 307 | 217 | 10 |
| Ribose | 9.55 | 307 | 217 | 10 |
|  |  | 307 | 103 | 10 |
|  |  | 320 | 191 | 12 |
| Rhamnose | 10.12, 10.33 | 277 | 189 | 17 |
|  |  | 219 | 75 | 15 |
|  |  | 219 | 130 | 15 |
| Mannitol | 11.86 | 319 | 157 | 12 |
|  |  | 319 | 129 | 12 |
| Sorbitol | 11.97 | 319 | 157 | 12 |
|  |  | 319 | 129 | 12 |
| Sorbitol-^13^C_6_ | 11.97 | 323 | 132 | 12 |
|  |  | 323 | 161 | 12 |
| Mannose | 12.72 | 319 | 129 | 12 |
|  |  | 319 | 157 | 12 |
| Fructose-^13^C_6_ | 11.9, 12.17 | 310 | 220 | 8 |
|  |  | 310 | 104 | 8 |
| Fructose | 11.9, 12.17 | 307 | 103 | 8 |
|  |  | 307 | 217 | 8 |
| Galactose | 12.0 | 319 | 129 | 12 |
|  |  | 319 | 157 | 12 |
| Glucose | 12.8 | 319 | 129 | 12 |
|  |  | 319 | 157 | 12 |
| Glucose-^13^C_6_ | 12.8 | 323 | 132 | 12 |
|  |  | 323 | 161 | 12 |
| Myo-inositol | 14.14 | 305 | 217 | 20 |
|  |  | 265 | 221 | 10 |
|  |  | 318 | 215 | 20 |
| Sucrose | 18.75 | 361 | 169 | 15 |
|  |  | 361 | 243 | 15 |
|  |  | 361 | 271 | 15 |
| Trehalose | 19.53 | 361 | 169 | 15 |
|  |  | 361 | 243 | 15 |
|  |  | 361 | 271 | 15 |
| Lactose | 19.73, 19.84 | 361 | 169 | 15 |
|  |  | 361 | 243 | 15 |
|  |  | 361 | 271 | 15 |
| Maltose | 20.32, 20.50 | 361 | 169 | 15 |
|  |  | 361 | 243 | 15 |
|  |  | 361 | 271 | 15 |
